# A simple computer vision pipeline reveals the effects of isolation on social interaction dynamics in *Drosophila*

**DOI:** 10.1371/journal.pcbi.1006410

**Published:** 2018-08-30

**Authors:** Guangda Liu, Tanmay Nath, Gerit A. Linneweber, Annelies Claeys, Zhengyu Guo, Jin Li, Mercedes Bengochea, Steve De Backer, Barbara Weyn, Manu Sneyders, Hans Nicasy, Peng Yu, Paul Scheunders, Bassem A. Hassan

**Affiliations:** 1 VIB Center for the Biology of Disease, VIB, Leuven, Belgium; 2 Center for Human Genetics, School of Medicine, University of Leuven, Leuven, Belgium; 3 Peira bvba, Turnhout, Belgium; 4 IMEC-Vision Lab, University of Antwerp, Antwerp, Belgium; 5 DCILabs, Keerbergen, Belgium; 6 Institut du Cerveau et de la Moelle Epinière (ICM) - Hôpital Pitié-Salpêtrière, Sorbonne Université, Inserm, CNRS, Paris, France; 7 Department of Electrical and Computer Engineering & TEES-AgriLife Center for Bioinformatics and Genomic Systems Engineering (CBGSE), Texas A&M University, College Station, Texas, United States of America; Imperial College London, UNITED KINGDOM

## Abstract

Isolation profoundly influences social behavior in all animals. In humans, isolation has serious effects on health. *Drosophila melanogaster* is a powerful model to study small-scale, temporally-transient social behavior. However, longer-term analysis of large groups of flies is hampered by the lack of effective and reliable tools. We built a new imaging arena and improved the existing tracking algorithm to reliably follow a large number of flies simultaneously. Next, based on the automatic classification of touch and graph-based social network analysis, we designed an algorithm to quantify changes in the social network in response to prior social isolation. We observed that isolation significantly and swiftly enhanced individual and local social network parameters depicting near-neighbor relationships. We explored the genome-wide molecular correlates of these behavioral changes and found that whereas behavior changed throughout the six days of isolation, gene expression alterations occurred largely on day one. These changes occurred mostly in metabolic genes, and we verified the metabolic changes by showing an increase of lipid content in isolated flies. In summary, we describe a highly reliable tracking and analysis pipeline for large groups of flies that we use to unravel the behavioral, molecular and physiological impact of isolation on social network dynamics in *Drosophila*.

## Author summary

Social isolation severely affects the behavior and physiology of social animals, including humans. The fruit fly is a powerful model for studying the mechanisms of development, health and disease and is also used to study social behaviors such as mating and aggression. However, these studies are limited to examining few individuals for shorts amounts of time, due to the lack of effective computational tools for the analysis of large groups over prolonged time. To overcome this hurdle, we built a new behavioral arena and developed new software that accurately tracks many flies simultaneously over long time periods. The arena is cheap and easy to build and the software works with low resolution videos. Using these improved tools, we studied social isolation in groups of male flies. We found that isolation caused flies to form stronger interactions with neighboring flies in their social network. These behavioral changes were preceded by transient changes in the expression of metabolism genes and eventually resulted in isolated flies accumulating fat, as has been previously observed in studies in mice and humans. Our study opens the door for the use of fruit flies in future studies of social isolation.

## Introduction

Social interactions range from simple touch contacts as seen in *Caenorhabditis elegans*, *Drosophila melanogaster* (fruit flies) and other animals, to the most complex social behaviors of primates including human beings [1–4]. Social interactions are a key feature of the life style of many species. In socially interacting species, isolation is thought to profoundly influence the response to stress, as shown in humans, monkeys, mice and fruit flies [5–10]. In humans, isolation is known to even affect the onset and progress of diseases [11]. Furthermore, a few studies revealed a potential link between social conditions and gene expression [12,13]. For example, Cole *et al* [14] profiled leukocytes from older humans with different social connections and found correlations between genome-wide transcriptional activity and social conditions, specifically the level of loneliness. This suggests that changes in social conditions, particularly the degree of “isolation”, may influence gene expression patterns.

To unravel the complex mechanisms of social behaviors, which occur in groups, tools are essential to analyze all interacting individuals at the same time. Whilst most studies still focus on dyadic interactions to limit the complexity of analysis [15–17], these studies cannot reflect the group-level relationships. More recently, researchers have begun to use social networks to analyze the relationships among the whole group of individuals [18–21]. Social network analysis allows to examine and quantify the social relationships in groups, such as the influence of individuals towards the whole group, spread of behavior throughout the group, etc. [22]. For our needs, social network analysis completes the toolset required to study the effects of isolation on group behavior, which includes also molecular and genetic analysis, and individual and grouped physiological and behavioral analysis.

*Drosophila melanogaster* is a very powerful genetic and neuroscience model that can to a certain extent readily be used to study social behavior and its underlying mechanisms. In order to study social behaviors in the fly, for example the effects of isolation on behavior and metabolism, a few key challenges remained open and needed to be addressed. These include appropriate experimental setups, reliability and accuracy of tracking, behavioral classification and detailed social network analysis. Presently, few of these are readily available with the required level of accuracy that we needed for our study on the effects of isolation on social behavior.

While there has been enormous progress in the study of pairs of flies in dyadic interactions [2], tools are lacking for larger groups. Especially, the efficient and accurate tracking of large groups of flies is a non-trivial problem. Ctrax, introduced by Branson and colleagues [23], was one of the first powerful algorithms to track multiple unmarked flies under laboratory conditions. While it can identify flies or other animals accurately when apart from each other in high resolution recordings, Ctrax creates tracking errors during interactions (loss and swap of individual identities and creation of new tracks) in a low resolution video [24]. Similar algorithms were used in 3D tracking methods using multiple cameras [25], in which the unexplained assumption was made that each interaction involves only 2 flies. Though such assumption may be valid for small numbers of flies, it may become incorrect as the number of flies increases. idTracker [26] was introduced to track multiple unmarked animals including flies to stop identity errors from propagating after interactions, even with complete occlusions. However, as stated by the authors, for idTracker to be in proper working order, the image of each individual should comprise at least 150 pixels, which is almost ten times more than that of a typical fly in videos acquired with easily accessible and affordable cameras. Therefore, the currently available tracking algorithms are either unreliable or they have too many constraints for simple and efficient usage, thereby making improvements like the one we propose in this paper necessary.

The next key issue in the analysis of social behaviors is the classification of various types of behaviors. Manual scoring is observer-dependent and can be arduous when multiple flies are involved [23]. Schneider J, *et al* [27] proposed a rule-based automated alternative to classify a number of well-defined interactions based on distance, duration and approach angle. However, the approach described in their study is not generic and cannot be applied to classify other behaviors. Also, the natural behavior of a fly is difficult to describe by a set of rules. An interactive machine learning based approach (JAABA) was recently proposed by Kabras et al [28], in which a user iteratively labels specific behaviors, and a machine learning algorithm uses these as training examples to predict future occurrences of the same behavior. However, the iterative process of creating the training dataset might be tedious for long videos. Thus, our approach differs from the above approach both in the classification strategy and in way that the training dataset is extracted.

Schneider *et al* [20] were the first to study social interactions of *Drosophila* at the group level by generating unweighted dynamic networks. Based on binary social network parameters, they concluded that social isolation had no effect on the networks. On the other hand, Simon *et al* [5] used a static parameter called the social space index, denoting the distance between resting non-interacting flies, to conclude that 7 days of isolation enlarges the social space between young (3–4 days old) flies. Our aim in this work is to extend these initial studies on social isolation, by investigating the effect of isolation on the social structure of flies at group-level over a long period of time.

Although a social interaction network can be useful to quantify social behaviors, it has its limitations when comparing different conditions, such as different biological repeats of an experiment. In that case, the intuition is to average the parameters of the social networks obtained from the individual repeats, which is only valid for a homogenous network structure [20]. This is certainly not true in our case, since in our social isolation experiments, each time a new batch of flies is used. In this paper, we propose a novel method to average social networks by matching individual flies, such that the variance of network parameters is reduced.

In summary, in this paper we describe an effective and reliable pipeline to image and analyze large groups of walking male flies. The pipeline consists of 1) an efficient and relatively low-cost acquisition setup, 2) an improved tracking algorithm (error-free for up to 16 male flies) based on a graph-theoretical framework, 3) a machine learning method for the classification of social interactions, and 4) a novel algorithm for computing an average social network to analyze the social structure. We apply this pipeline to study behavioral changes due to isolation and correlate these with changes in genome-wide gene expression. Our results show strong and specific effects of isolation on social network dynamics, metabolic gene expression and animal physiology.

## Results

### Data acquisition and tracking of large groups of fruit flies

**Fig 1:**
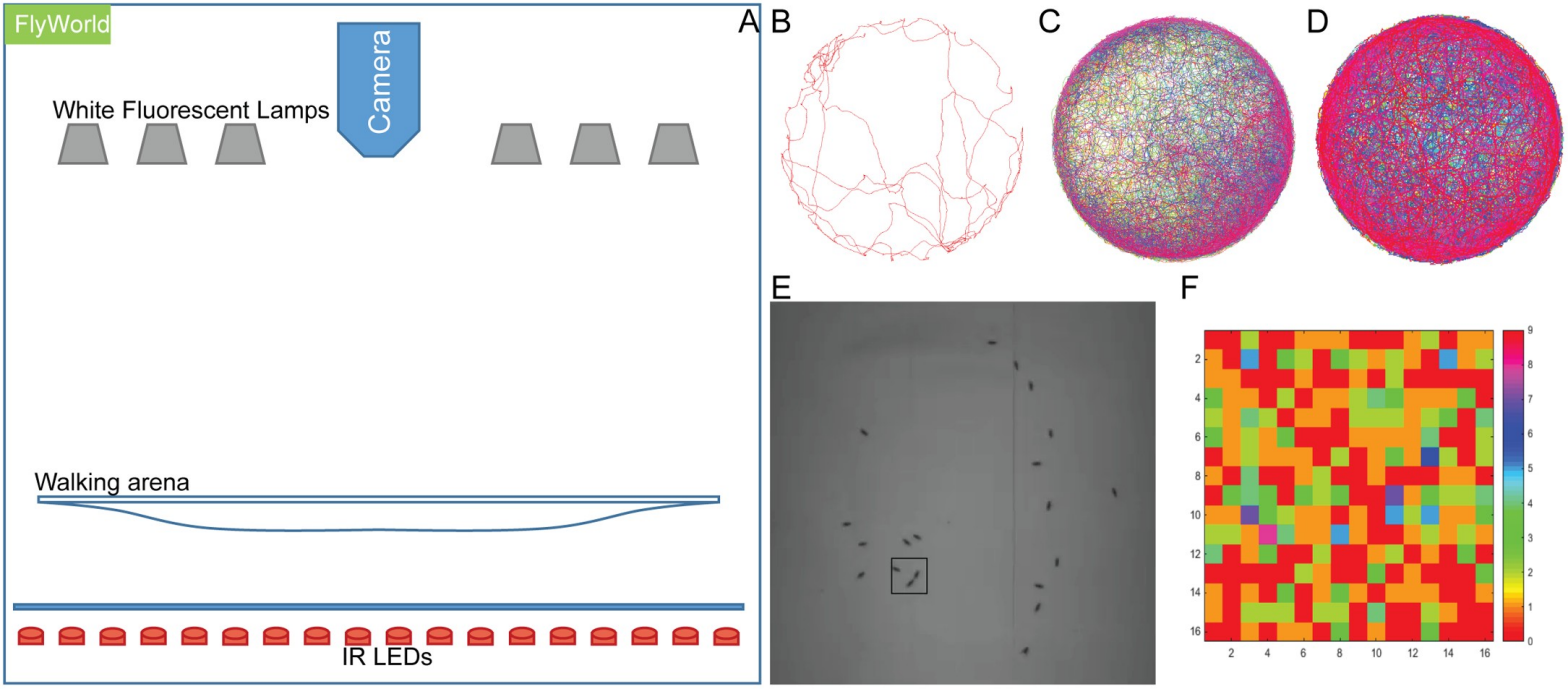
Tracking and behavior classification of flies with Flyworld and Flytracker. (A) Schematics of Flyworld, with camera, illumination system, and walking arena. (B-D) Example trajectories of different numbers (1, 16 and 50) of male flies tracked by Flytracker. (E) A touch event where one fly’s head approaches another’s tail in darkness, recorded with an infrared camera. (F) Adjacency matrix of touch behaviors. Colors represent frequencies of touch behavior. Mathematically, no self-interaction was defined.

Data acquisition was performed using Flyworld, a closed computer vision setup, consisting of a specifically designed arena [29] and a high speed digital camera (Fig 1A). We previously developed Flytracker [30], an algorithm to track fruit flies in a video using a graph-theoretical framework that ensures an accurate estimate of the number of flies, even when multiple flies interact. As a result of the algorithm, we obtain trajectories for each fly’s body center along with its head and tail positions. Flytracker was able to track up to 50 male flies without further error-fixing (Fig 1B–1D and S1–S3 Videos).

To demonstrate the improvement Flytracker provides compared to previously available methods, we used Ctrax to reanalyze our videos, and found that it detected wrong numbers of flies in 60–70% of the frames (Table 1, S4 Video), varying from 6 to 17 flies in 16-fly videos. This resulted in broken tracks and thus identity losses, requiring further patching effort to connect the broken tracks. Next, we tested the resolution limit of Flytracker. To this end we recorded videos of male and female flies walking in an arena originally designed for Buridan’s paradigm [31] (see S1 File for a detailed illustration of the setup). Briefly, our Buridan’s arena consists of a 12cm platform surrounded by water preventing the flies form reaching an illuminated cylinder beyond the water. Flies with clipped wings are placed in the area and are filmed from above at low resolution (7–10 pixels per fly) as they walk freely within the arena. We tested the tracking of 1, 2 and 10 flies. In all cases Flytracker successfully tracked the flies (S5 Video) as they walked within the arena. However, tracks were lost when the flies were at the very edge of the arena touching the water, likely due to changes in pixel intensity at the arena-water edge. These data suggest that Flytracker can successfully identify and track individual flies or group of flies in videos form multiple settings and at a range of resolutions, including as low as 7–10 pixels per fly.

**Table 1:** Tracking results of Ctrax and Flytracker with different numbers of flies and videos at different frame rates.

Videos	Frames analyzed	Frame Rate	Number of flies	Frames with incorrect number of flies detected (%)		Tracks detected	
				Ctrax	Flytracker	Ctrax	Flytracker
1	52347	15	16	68.01	0	1050	16
2	106198	30	16	48.68	0	840	16
3	27199	60	16	75.83	0	549	16
4	27060	120	16	72.57	0	482	16
5	27189	300	16	98.9	0	677	16
1	28480	30	10	48.426	0	337	10
2	31829	30	16	82.43	0	366	16
3	9381	30	32	92.12	0	780	32
4	29105	30	50	43.1	0	137	50

To validate Flytracker, we established ground-truth. For this, we manually tracked all flies, and found only three identity swaps in one 50-fly 1-hour video and zero errors in two randomly chosen 16-fly 1-hour videos (S6 Video, details are described in the Methods). The swaps of identities in the 50-fly video occurred when 2 flies jumped while crossing each other. In all subsequent experiments, groups of 16 male flies were used to ensure that no tracking errors occur, which is crucial for the following procedures.

### Detection of touch interactions between flies

After video acquisition and tracking, a supervised machine learning-based method as described by Nath *et al* [32] was used to detect the social interactions between male flies. Social interactions were defined as close physical contacts between two or more flies. In this paper, only “head to-tail touch” events (see Methods for a detailed definition) were considered (Fig 1E, S7 Video). A “touch” was defined as a head-to tail touch event in a single frame. A “touch interaction” is obtained when a touch lasts for at least 15 consecutive frames (~0.5s). A gap of at least 15 frames between two consecutive interactions was required to separate the two interactions.

A training and test dataset was generated by a human expert, annotating frame-by-frame touch events in the video. Using the fly trajectories and body positions, relative and temporal features were extracted. Based on these features, the supervised learning algorithm was trained to recognize single frame head-to-tail touch events after which touch interactions could be extracted.

### Social network construction

From each tracked video, a directional weighted interaction network for touch interactions was constructed. This network can be represented by an adjacency matrix. Fig 1F shows such a matrix as a heat map. A sample adjacency matrix and its corresponding network are shown in S6A and S6B Fig. Each node represents a fly and each edge denotes that there have been one or more touch interactions between two flies, where the thickness of the edge indicates the number of interactions.

From multiple repeats of an experiment under the same conditions, an average network is calculated by first matching the individual social networks using a graph matching technique, followed by averaging the weights of these matched graphs in order to ensure optimal correspondence between flies at similar locations across the 10 networks [33] (S2 Fig).

### Social parameters

The average weighted directional networks of control experiments and experiments under social isolation conditions were compared quantitatively. For this, we calculated three types of parameters: individual fly parameters such as the Weighted Degree (describing the tendency of a fly to interact), local interaction parameters such as the Clustering Coefficient (describing the strength of interactions between neighboring flies) and global interaction parameters such as the Global Efficiency (describing the overall structure of the network). The key parameters for our analysis were the Weighted Degree, Weighted Total Interactions (describing the tendency of flies to interact in the whole group), Clustering Coefficient, Global Efficiency, Assortativity (describing the tendency of interactions between similarly active flies), Betweenness Centrality (describing the importance of a fly as connection “hub” to other flies) and Transitivity (describing the global tendency of flies to interact with another fly with shared adjacent flies). For a detailed description of all the parameters please refer to the methods section. Besides these network parameters, the Total Walking Distance is calculated directly from the tracks as a measure for the locomotor activity.

### Effects of isolation on social network structure

**Fig 2:**
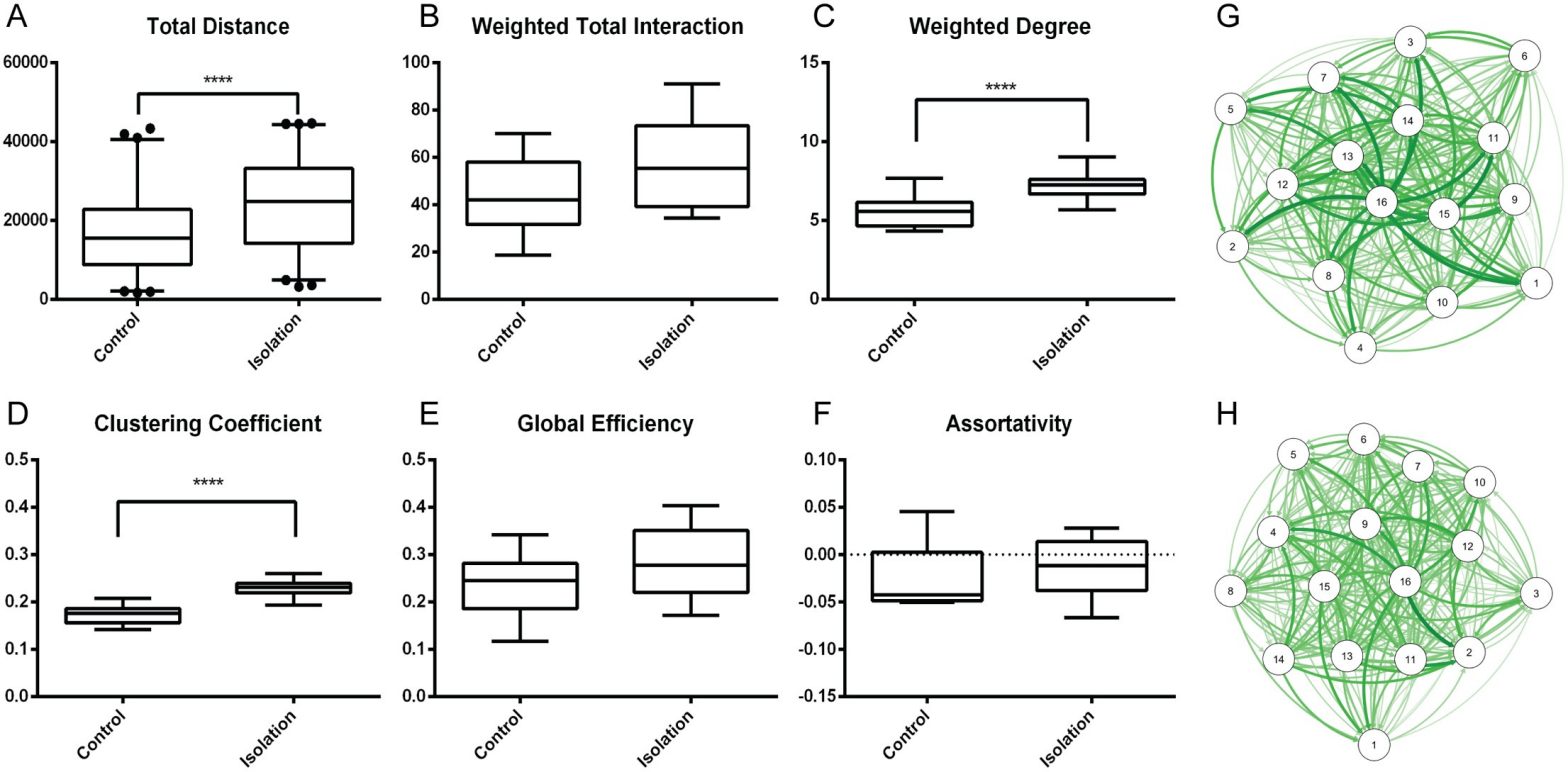
Effects of social isolation on the parameters of average networks. (A) The Total Walking Distance in the isolated group is significantly higher than in the control group (p<0.0001). (B) No significant difference is found between the Weighted Total Interaction of the control and isolation groups. (C and D) A significant increase of Weighted Degree and Clustering Coefficient was observed between average networks of control and isolation groups (p<0.0001). (E and F) No significant difference was found between the global parameters Global Efficiency and Assortativity of the control and isolation groups. Average networks representing 10 repeats of experiment on control (G) and isolation (H) groups. All plots use 2.5th to 97.5th percentiles as whiskers and 25th to 75th percentiles as box, with the median value in the middle of the box (n = 10).

In order to test our social network analysis pipeline and to study the effect of isolation on the social network structure, we performed isolation experiments. In a first set of experiments, we tested the effect of 6-day isolation on the social network. From ten repeats of the experiment, each time with a different control and isolation group, we constructed the average networks of the control and isolated groups and analyzed their network parameters (Fig 2G and 2H).

### Locomotor activity and social interactions

A strong effect of 6-day social isolation was that the flies displayed an enhanced locomotor activity, demonstrated by a significant increase in Total Walking Distance (Fig 2A). However, when looking at the global network characteristics, e.g. by the global network parameter Weighted Total Interaction, no significant differences between isolated and control flies were found (Fig 2B). This indicates that the total social activity across the entire network was not significantly affected by 6 days of isolation. In contrast, we found a significant increase of individual network parameters such as the Weighted Degree and the other degree related parameters for the isolated flies (Fig 2C, S3A–S3C, S3E and S3F Fig). Thus, while the total number interactions remained similar, individuals within the isolated group revealed an increased amount of interactions and an enhanced locomotor activity.

### Local versus global social parameters of the network

To address the observation that isolation enlarges the number of interactions without a major change in the global structure of the social network, we examined local network parameters. No significant difference between control and isolated flies was found in the Betweenness Centrality. Therefore, no evidence was found for an enhanced activity of specific “hub” flies (S3D Fig). In contrast, the Clustering Coefficient of isolated flies was found to be significantly higher than that of controls (Fig 2D), indicating a significant increase in the amount of local interactions among neighboring flies within the network. In other words, we observe an increase in the amount of social interactions among local groups of isolated flies relative to controls.

As stated above, the activity at the whole-group level was not affected by isolation, since along with the Weighted Total Interaction, the other global network parameters did not differ significantly between the control and isolated flies. This was the case for Global Efficiency, Density, Assortativity and Transitivity, respectively (Fig 2E and 2F and S3G and S3H Fig).

In summary, we found that 6 days of isolation caused an increase in the locomotor activity and frequency of interactions at the local level without changing the global structure of the social network.

### Temporal dynamics of social network structure upon isolation

**Fig 3:**
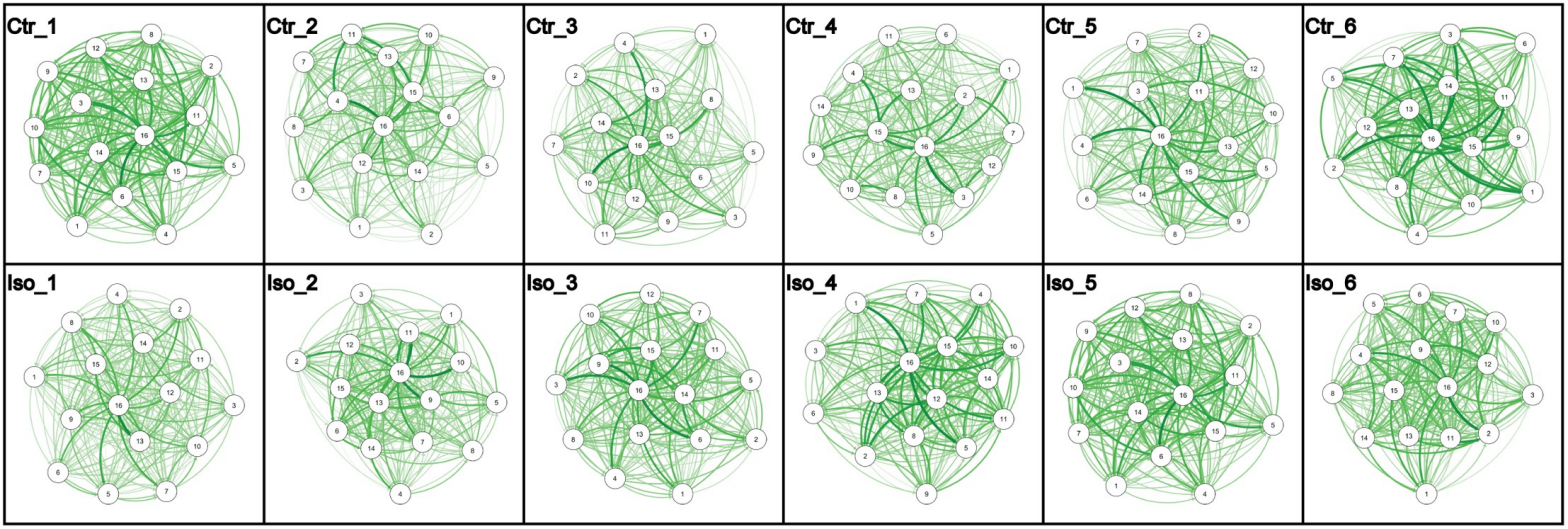
The change of average networks of the control and isolated groups with changing isolation time interval. Ctr_1 to Ctr_6 are averaged networks from flies in the control group from 1 to 6 days of preparation. Iso_1 to Iso_6 are averaged networks from flies in the isolation group kept in isolation from 1 to 6 days. Each average network is the mean graph of the networks from 10 biological repeats. Nodes represent “average flies” and edges represent the average number of interactions from one “average fly” to another. Isolated flies generally display more interactions than control flies.

The isolation period of 6 days may be longer than required to cause changes in social behavior. Conversely, behavioral changes may have occurred and then disappeared again within the 6-day period. To determine the minimum number of days of isolation required to result in social behavioral changes, we repeated the isolation experiments for periods of 1, 2, 3, 4 and 5 days of isolation using the same methodology. Fig 3 shows the obtained average networks. Interestingly, compared to the control groups, the visualization of the networks of the isolated flies shows increased connectivity (which amounts to an increase in the number of interactions, proportional to the thickness of the arrows), which rises steadily and rapidly over time.

**Fig 4:**
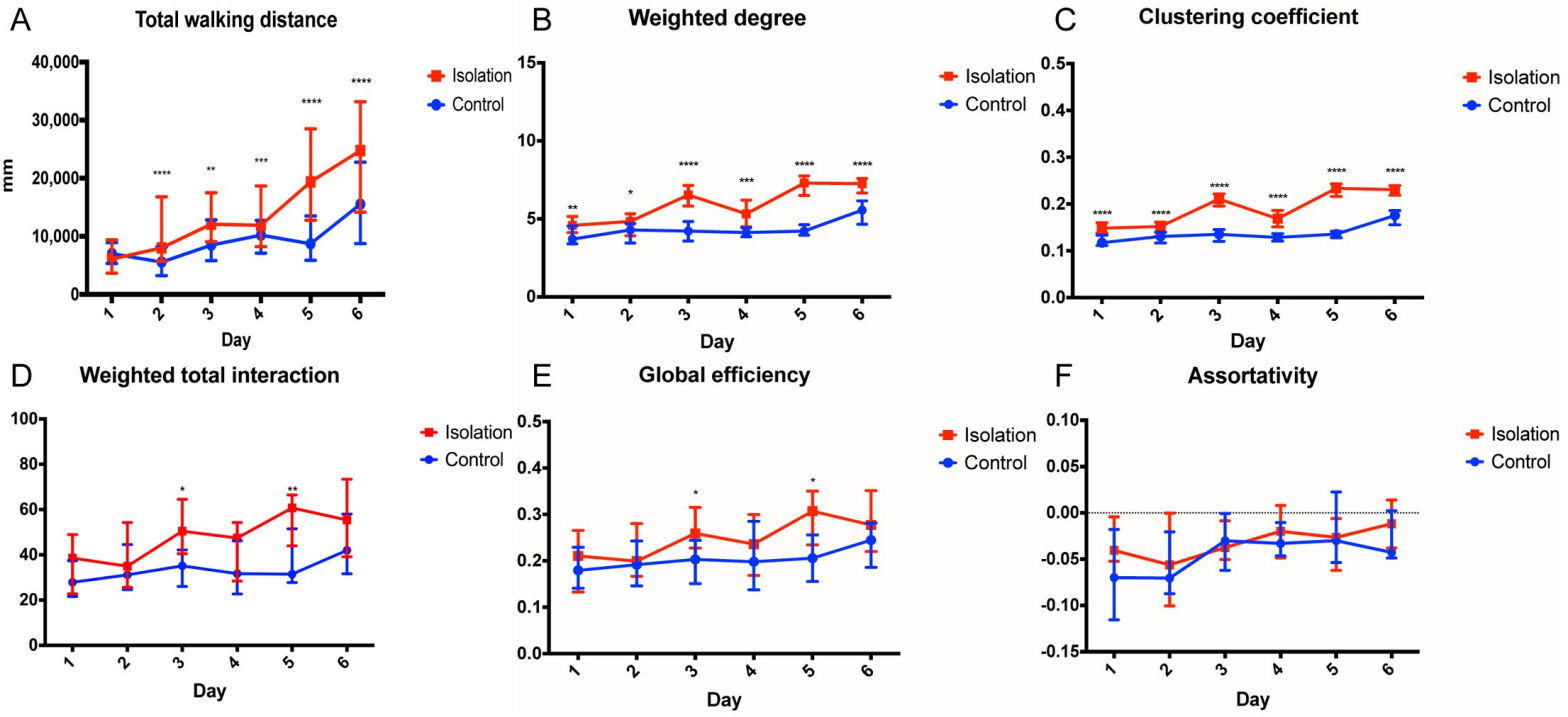
Changes of locomotor activity and social network parameters with changing isolation time interval. (A) The Total Walking Distance, represented by its median with an interquartile range, shows significant differences between control and isolation groups (p<0.0001) except after 1 day of isolation. Different parameters reveal differences between control (blue) and isolated (red) groups over time. Significance is denoted with asterisks, the number of which represents the negative order of magnitude of the p value. Data are shown as median values with interquartile ranges, with 10 repeats on each isolation day (n = 10). (B) Individual parameter Weighted Degree of control and isolated flies shows a significant difference from day 1 which increases significantly after day 2. (C) Difference in the local parameter Clustering Coefficient also shows from day 1 and increases over 6 days. (D) Global parameter Weighted Total Interaction significantly differs only on day3 and day5 and not on other days. (E) Global parameter Global Efficiency significantly differs on day3 and day5 only. (F) No significant difference was found on the global parameter Assortativity.

Isolated flies walked significantly more than flies in control groups starting from day 2 of the experiment onwards (see Fig 4A). The walking distance continued to rise steadily during the 6 days of isolation. Similarly, an increase in Weighted Degree and Clustering Coefficient occurred very early (day 1) and persisted over the entire isolation period (Fig 4B and 4C). These results indicate that an increase in locomotor activity, individual interactivity and amount of local interactions occurs very quickly after social isolation, with a persistent long-lasting effect. In contrast, we found no significant differences in global parameters. Weighted Total Interaction and Global Efficiency showed an increase on days 3 and 5, but not on other days (Fig 4D and 4E). We found similar effects for the other global parameters Assortativity, Density and Transitivity (Fig 4F and S4G and S4H Fig).

### Social isolation and genome-wide gene expression

The network data indicated that individual and local social parameter changes after social isolation are early-occurring and long-lasting. Next, we investigated if these behavioral changes were accompanied by alterations in gene expression [34]. To this end, we performed RNA-sequencing of 16 male flies isolated for 1, 2, 3 and 6 days, and their equally treated grouped housed control flies.

**Fig 5:**
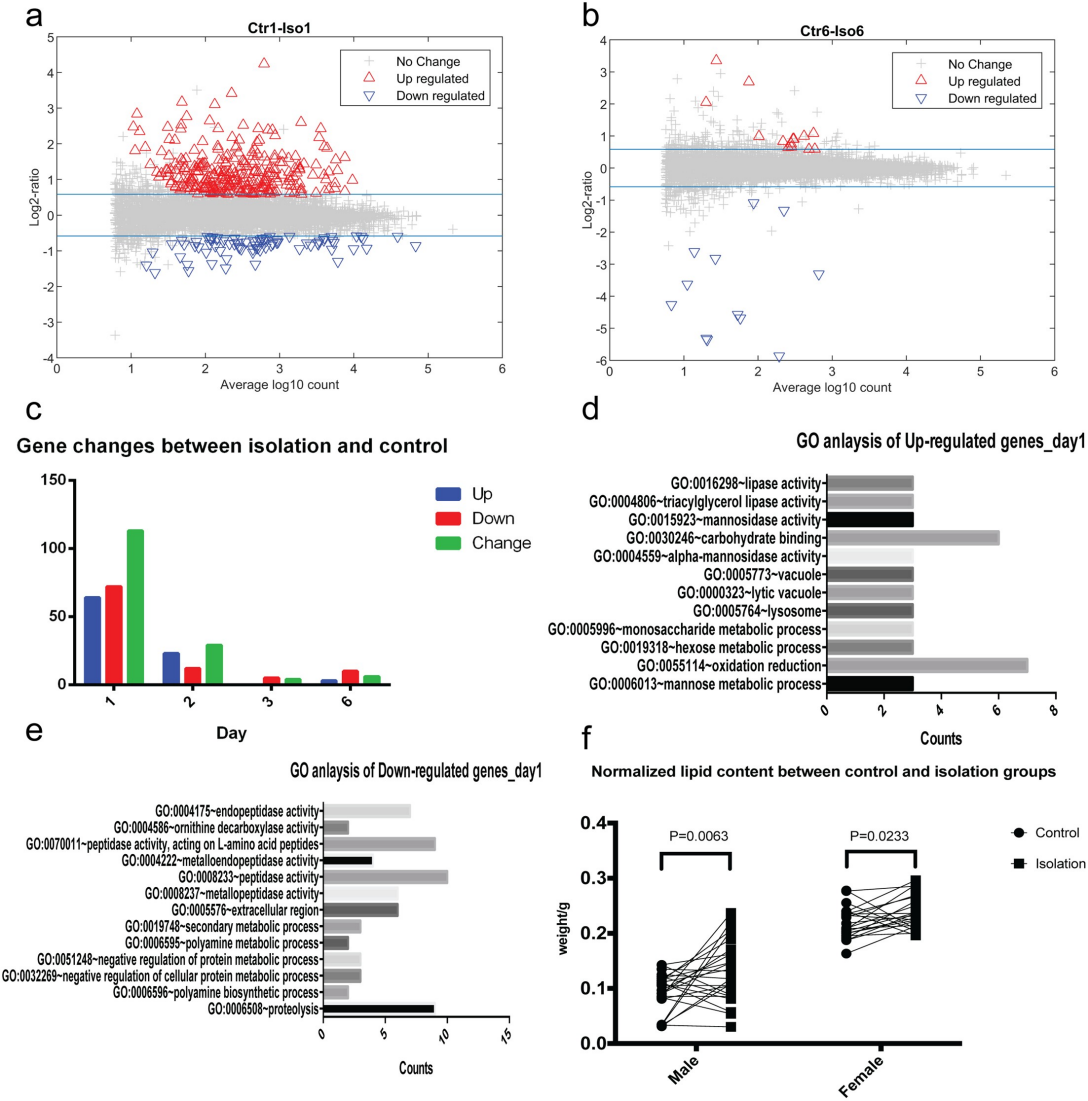
Genome-wide gene analysis of expression and physiological changes. (A, B) Scatter plots of log_2_ of fold changes versus log_10_ of gene counts on day 1 and day 6. Red up triangles show genes whose expression levels are significantly increased more than 1.5 folds. Blue down triangles show genes whose expression levels are significantly decreased more than 1.5 folds. Blue lines show the ±*log*_2_ 1.5 thresholds. (C) Plot of the number of genes whose expression is upregulated (blue), downregulated (red), or changed either way (green) specifically between isolated and control groups on Days 1, 2, 3 and 6. All experiments were repeated 3 times. (D, E) Gene Ontology analysis of differentially expressed genes on day one. Most changed genes on day one has been clustered into metabolism related categories, in both up- and down-regulated genes. (F) Lipid/dry-weight changes between control and isolation groups in male and female flies. All groups are paired and paired t-test show significant increase in lipid/dry-weight proportion in isolated flies than control male flies (n = 25, P = 0.0063). Isolated female flies also have increased lipid proportion to dry-weight than control flies (n = 22, P = 0.0233).

Our differential expression analysis showed that the vast majority of changes in gene expression occurred after 1 day of isolation and that the changes were very transient (Fig 5A). A few changes were seen on Day 2 (Fig 5B), and the effects on gene expression almost completely disappeared on Day 3 and Day 6 (S5A and S5B Fig). Specifically, 136 genes showed significant changes in expression levels on Day 1, with 64 upregulated genes and 72 downregulated genes (Fig 5C). On Day 2, there were 23 upregulated genes, including 7 persisting from Day 1 and 12 downregulated genes, all persisting from Day 1. Thus, while both behavioral and gene expression level changes were initiated early (Day 1 and/or Day 2), behavioral alterations were long lasting, while changes in gene expression levels were transient.

Altogether, our data so far suggest that already after only a single day of isolation flies alter their physiology and behavior to promote social interactions with neighboring flies. One of the individual fly parameters that changes early and persistently is walking distance. We therefore asked how this individual fly parameter might relate with local network-level parameters affected by isolation such as Weighted Degree and Clustering Coefficient and how this might evolve over isolation time. To this end, we calculated the correlation coefficient between walking distance and local interaction parameters on days 1, 2 and 6. We find that after 1 day of isolation there is a strong correlation between walking distance and local interaction parameters in isolated flies but not in controls. These correlations emerge in controls on day 2 and persist in both groups on day 6 (S7 Fig). This suggests that already after one day of isolation flies dedicate much of their walking to social interaction, even when there is not yet a significant change in the total distance they walk.

Finally, to gain insight into the physiological processes affected by isolation-induced that might trigger early changes in behavior, we analyzed the isolation-induced gene expression alterations. The DAVID Bioinformatics Resources 6.8 (Beta) [35,36] was used to investigate the functions of those genes using the GO (Gene Ontology) terms: MF (Molecular Function), CC (Cellular Component) and BP (Biological Process). GO enrichment analysis shows that most changes affect genes involved in metabolism (Fig 5D and 5E and S5C and S5D Fig). Specifically, there was enrichment for genes involved in lipid (lipase activity) and carbohydrate metabolism and cellular degradative processes among the upregulated genes, and enrichment for genes involved in amino acid and biogenic amine metabolism among the downregulated genes. No other categories were found to be significantly enriched.

Next, we investigated if isolation affects gene expression by changing isoform balance through alternative splicing. To this end, alternative splicing events in our RNA-seq data [37] were profiled. However, a very limited number of alternative splicing events (S1 Table) was found, affecting no more than 1 or 2 genes on any given day of isolation. On early days (Day 1 and Day 2) genes encoding cytoskeletal proteins, such as *Myofilin*, coding a core protein in the thick filaments of muscle [38], and *Pif1B*, a Janus Kinase and microtubule-interacting protein coding gene [39], were affected. *Mf* was alternatively spliced on both the first two days. After 3 days of isolation, a gene encoding a nucleic acid regulating protein, namely the Purine-nucleoside Phosphorylase Related protein, *CG16758* [40], and the *squid* (*sqd*) [41] gene encoding an RNA-binding protein were affected. All these changes involved a reduced number of exons in the mature mRNA. Interestingly, in flies isolated for 6 days, the *desaturase 1* (*desat1*) gene, whose product is involved in fatty acid synthesis [42,43] and pheromone (cuticular hydrocarbons) biosynthesis [44] was affected through the choice of an alternative first exon.

### Social isolation and lipid content changes

Our genomic-wide expression analysis showed that isolation of fruit flies caused changes in the expression and splicing of genes involved in lipid and hydrocarbon metabolism. To test if these changes are biologically relevant, we tested if the observed changes correlate with physiological changes *in vivo* by measuring the weight and lipid content of isolated flies compared to controls. The wet weight, dry weight, and lipid weight of 16 flies of each group were measured (S5E–S5G Fig), hence lipid weight as well as lipid weight normalized by dry weight (lipid/dry-weight) were calculated (S5H and S5F Fig). We found that isolated male flies, have significantly higher lipid content (13.36% ± 5.52%, *mean* ± *SD*) than that of control flies (9.40% ± 3.54%, *mean* ± *SD*) (Fig 5F). Furthermore, the absolute values of dry weight and ether weight are both significantly higher in isolation group, whereas lipid extracted weight has less difference compared to absolute lipid weight. This suggests the absolute dry weight increase in isolation group comes mainly from lipid composition. Additionally, we observed a similar increase in lipid/dry-weight was in isolated female flies (Fig 5F).

## Discussion

In this paper, we describe the establishment of a novel framework for the accurate tracking of multiple flies, their behavior classification and a method to study social network dynamics. We use this framework to study the effects of isolation on social network dynamics and performed the first genome-wide gene expression analysis to correlate transcriptomic and behavioral changes upon social isolation in male *Drosophila*. Importantly, this pipeline uses easily available hardware to record long videos of interacting flies. The use of a graph-theoretic structure for the tracking algorithm allows to detect a constant number of flies in each frame, thus it is highly reliable and accurate and no further steps are required to correct for broken tracks. We confirmed this by the manual tracking of 2 randomly chosen videos. The supervised machine learning approach that we used for classification is generic and allows to extend the study of mere “touch” interactions to other types of interactions such as “sniff” or “chase”. Therefore, this platform is well suited for an accurate large-scale, long-term assessment of social behavior in flies. In theory, this approach is transferrable to any other species. Additionally, the interfaces between different components of the pipeline are compatible with other established methods, so that other researchers can adapt their experiments to our pipeline with minimum effort.

In this study, we implement for the first time the use of an average network for social touch interactions for a large group of male flies. Previously, [20] work used social network analysis to study group behavior in flies. In their research, the dynamic iterative network was built based on the consecutive interactions among the individuals, for example 33 consecutive interactions were used for iterative network construction in most results, rather than a fixed period of interactions. When they compared the differences between networks, they averaged the parameters of networks, which might lose information of heterogeneity within the network since the flies are not in similar position/functions within the group. Unlike network analysis in other fields [45–47], individuals (flies) in our research are not the same across networks, which could create hierarchy within the group. Taking the heterogeneity of network into account, matching individuals across the networks while studying multiple network is crucial for accurate analysis of behaviors. The average network built in our work based on graph matching of individuals first minimized the variance across the networks hence generates closer result to reality.

Having developed these tools, we applied them to study the consequences of isolation on social network dynamics. The results showed that social isolation affects the interactions between isolated flies by increased locomotion activity and local social interactions, with minimal effects on the global pattern of social interactions.

Social isolation in humans is a risk factor for, and may contribute to, poorer overall cognitive performance and executive function, increased negativity and depressive cognition [48]. There are more and more studies trying to unravel the relations between social environment changes, for example, isolation, and gene expression, to understand the process of the social influence. Previous studies on leukocyte of elderly human being have revealed some connections between social loneliness to immune system gene expression changes [14]. A study on mice shows [49] showing upregulated brain-derived neurotrophic factor upon social isolation. We tracked the changes in gene expression over social isolation time and found that changes in gene expression occurred very early together with, and even before some of, the behavioral changes. These gene expression alterations were highly specific, affecting mainly metabolism, and very transient. Further experiment demonstrated that isolation has caused increase in the weight of *Drosophila* compared to that of control group, both in the gross weight (wet weight, dry weight, and ether weight) and normalized lipid content. It is tantalizing to speculate that a transient alteration in the metabolic state of flies upon social isolation plays a potentially causal role in long term behavioral changes, but this needs to be studied in much greater depth in the future.

## Methods

### Fly maintenance and rearing

Canton-S wild-type *Drosophila melanogaster* were raised on standard cornmeal/agar medium in large vials at room temperature (22 °C) and a 12h light/dark cycle. 9-day old flies were taken out of their vials and put onto a petri dish after cold anesthetization. Male flies were collected under a stereo microscope. Groups of 16 male flies were sorted and introduced into a new small vial. All flies were kept in new vials for one day before treatment.

### Experimental isolation procedure

After one day, the flies were transferred to new small vials either individually or grouped depending on the treatment. For the isolation group, 16 flies were transferred to 16 new small vials and kept in the small vial for 1–6 days, depending on the length of isolation. For the control groups, all 16 flies were transferred to one small vial and kept for 1–6 days, depending on the length of treatment in the experimental group. In total for each repeat of the experiment, there were 6 groups of isolated flies and 6 groups of control flies. All experiments were repeated 10 times.

### Data acquisition in the large group walking arena Flyworld

We constructed Flyworld (Peira bvba, Turnhout, Belgium) to record multiple flies in a walking arena [29] under different illumination conditions, with a high-speed infrared camera (Fig 1A). The 3D models of the arena and key supporting materials (S1 File) are developed using SolidWorks 2012 which can be read and measured using a free version software, eDrawing. The IR LED panel underneath the arena was made by Peira. The main parts including arena and camera cost approximately 3000 euros. On the day of recording, flies were taken out of vials and put into the fly chamber of Flyworld using cold anesthetization 2–4 hours after the start of light cycle. After a one-minute recovery period, the locomotion activities of the flies were recorded for 60 minutes in complete darkness.

### Video acquisition in Flyworld

Movies were recorded at 30 fps using a high-speed infrared Genie HM640 camera (640x480px, Teledyne Dalsa, Waterloo, Canada). The recording chamber was designed to enforce the movements of flies in a two dimensional plane [29]. In order to accomplish this, the height of the chamber was limited to 3.5 mm, which prevents flying but still permits free walking without clipping off the wings [23]. The ceiling of the chamber was coated with Sigmacote (Sigma-Aldrich NV/SA, Diegem, Belgium), which prevents flies from walking on the ceiling and obscuring the imaging of other flies. This procedure is crucial to achieve error free tracking, since it reduces the chance of total occlusion between flies. The chamber and the cover were cleaned with 75% ethanol between each experiment to remove possible residues on the setup. The videos were recorded in an uncompressed format, and were later compressed using MPEG-4. A compression rate was chosen which does not compromise the image quality but reduces the video size.

### Flytracker a reliable tracker for multiple walking flies

The Flytracker is developed mainly using MATLAB (version 2015b, tested on both Mac OS Sierra and Windows 7 systems, now available for download and development at github (https://github.com/flytracker/flytracker). Flytracker’s reliability in tracking multiple flies is based on one particular assumption that shapes the algorithm: the number of flies remains constant throughout the video. Since the field of view of the camera in Flyworld covers the whole walking arena, this assumption is automatically met. As in Ctrax [23], tracking is achieved in two steps: fly detection and identity assignment.

During fly detection, each frame is segmented to separate flies from the background. Each fly is detected as a set of white connected components on a black background. Each fly in this setup has an area of approximately 20 pixels. The key feature of Flytracker is a graph-based framework [50] to estimate the number of interacting flies in a frame. If the number of connected components is lower than the total number of flies in a frame, one or more connected components in a frame will contain 2 or more interacting flies. These are easily detected since their sizes are larger than the typical size of a single fly.

When assuming that the previous frame has been processed and all flies are identified, a bipartite graph is constructed between the previous and the current frame. A bipartite graph contains 2 disjoint subsets of nodes, with edges only connecting one subset to the other [51]. In our case, the first subset denotes the previous frame and contains all individual flies as nodes while the second subset denotes the current frame and initially contains all connected components that contain 2 or more interacting flies. These nodes are then duplicated n times, where n is the number of missing connected components, i.e. the total number of flies minus the number of connected components in the frame (S1A Fig). Edges connect the nodes from the previous frame with the nodes from the current frame, and are weighted by the distance between the locations of the corresponding flies. The assignment problem has now become a minimum weighted matching problem and is solved using the Hungarian Algorithm [52]. As a result, a minimum weighted graph is obtained in which each node from the previous frame is connected to one of the nodes of the current frame. The number of edges that a node in the current frame receives is then the number of interacting flies in the corresponding connected component. This connected component is then split into individual flies using the Expectation-Maximization algorithm, also applied by Ctrax. The main advantage of the proposed graph-based method is that in each frame the correct number of flies and thus tracks is always identified.

Once all connected components are split to contain one unique fly, the flies can be identified by a unique assignment with the flies from the previous frame. For this identity assignment step, exactly the same framework is applied using a weighted bipartite graph where one set of nodes in the graph represents the flies in the previous frame, while the other set of nodes represents the flies in the current frame, again connected by an edge, weighted by the distances between the corresponding fly locations. Minimization of this weighted bipartite graph is again done by the Hungarian algorithm [52,53]. The orientation of the flies is determined based on the assumption that a fly mainly walks with its head pointing in the direction of its motion [23].

### Manual validation of tracking results

To validate the tracking results, two experts have independently examined one 50-fly and two 16-fly 1-hour videos. In Supplementary Video 4, a particular fly (Fly 1) is labeled with a black box for easy tracking. Positions of the particular fly, 30 frames before and after the current frame are displayed in black dots to help the observer to predict possible losses of identity. All flies were labeled with their fly ID number. During each validation cycle, an observer followed the particular fly throughout the whole 1-hour video to check if there is a loss or swap of identities. After completion of one cycle, the observer choses another particular fly (Fly 2) and so on to repeat the above procedure until all flies have been analyzed. The same procedure was applied to validate two 16-fly 1-hour videos.

### Classification of social interactions in the tracking results

Once all flies are tracked, social interactions between the flies can be detected. Flies can exhibit different types of interaction (e.g. chasing, sniffing, …). Given the low spatial resolution of Flyworld, detailed fly body kinematics are not visible, which limits our search to simple types of interactions. In this work, we focused on one particular touch event, in which one fly (the interactor fly) approaches another fly (the interacted fly) by a head-to-tail contact. A touch interaction was then defined as a situation where a touch event lasts for at least 0.5 seconds, while a gap of at least 0.5 seconds was required to separate two distinct touch interactions.

Single frame touch events were determined by a supervised learning method, using Support Vector Machines (Nath *et al* [32]). For this, a number of features was calculated from the fly tracks. In order to be able to distinct a single frame head-to-tail touch event from other touch events, these features need to describe the specific action dynamics from the fly. For this, 22 temporal features are defined, i.e. the Euclidean distances between the interactor and interacted flies over 11 time frames centered around the time frame under consideration, and the relative displacements of the interactor fly over the same time window.

A training data set was built by manually annotating touch events. The training data set contained 1000 frames of touch events and an equal number of no-touch events. Testing data sets were built by manually annotating 3 different fly-pairs exhibiting the two events. Each testing data set contains 1000 events, to be classified into one of the two behavioral classes. The result of the classifier was validated with the ground–truth of the testing data set. A separate comparison was also made with a second expert’s annotation on the same video and on the same set of flies.

Once all frames are classified either as a touch event or as a no-touch event, the touch interactions are determined based on the 0.5 second rule. All fly trajectories are then subdivided into intervals in which the fly either exhibits a touch interaction or not.

### Construction of a social interaction network

After video annotation and classification, the results are represented by a weighted adjacency matrix, which is a square matrix, with each element denoting a particular fly pair. The value of the element (weight) represents the number of touch interactions between the particular fly pair. From the adjacency matrix, a social network is constructed, as a directed weighted graph, by representing flies by nodes and their specific touch interactions by edges. An illustrative example adjacency matrix and its corresponding social network is shown in S6A and S6B Fig. The sample adjacency matrix contains the number of touch interactions between any pair of flies. Each node in the corresponding social network in S6B Fig represents a fly and an edge denotes that the particular fly pair exhibits touch interactions. Each edge is weighted by the number of touch interactions between the fly pair, as shown by the thickness of the edge.

The network is directed in order to identify the principal interactor fly and the interacted fly: the edge starts from the node of the principal interactor fly and points to the node of the interacted fly. In the networks, the flies are labeled with numbers (from 1 to 16) in ascending order of out-going activity (number of touch interactions as an interactor fly) so that all flies with similar network ranking across different networks are placed into similar positions.

### Quantification of the social network by network parameters

Each social network was quantified by nine well established network parameters, as previously defined by Wey. T *et al* [21]. Each adjacency matrix is normalized by its maximum weight before computing the social parameters. We use the same example network from S2 Fig to explain and compute the parameters. S2 Table show the values of all parameters of the sample social network. The parameters are subdivided into three categories.

Individual parameters:

1 In-Degree, Out-Degree, Degree. These degree-related individual parameters are directional parameters. The Out-Degree is the number of edges that leave a node, while the In-Degree is the number of edges that reach a node. In the sample social network, fly X1 interacts with flies X2, X3, X4, X5, and X6, leading to an Out-Degree of 5. Fly X1 receives interactions from flies X2, X3, and X5, so its In-Degree is 3. The Degree is the sum of the In-Degree and Out-Degree.
2 Weighted In-Degree, Out-Degree, and Degree. When accounting for the weights of the edges (i.e. the actual number of touch interactions between the nodes), weighted values for the degrees are obtained.

Local parameters:

3 Clustering Coefficient: This parameter indicates how interactive the neighbors of a node are. A node with high clustering coefficient has a high number of connected neighbors that are also connected to each other. The Clustering Coefficient for a weighted directed network is defined by Fagiolo. G. *et*.*al* [54] and is computed using the Brain connectivity toolbox (BCT) [55].
4 Betweenness Centrality [56]: Betweenness Centrality is a measure of the number of shortest paths passing through a node. A node with high Betweenness Centrality acts as a pivot in the network information flow. Betweenness Centrality of a weighted directed graph is computed using BCT.

Global parameters:

5 Assortativity: This parameter indicates the preference of nodes in a network to connect to other nodes that have similar degree [20]. In an assortative mixing network, nodes with similar degree preferably connect, while in a dissortative network, nodes with low degree tend to connect to nodes with high degree, which makes the network less homogeneous.
6 Global Efficiency: This parameter is the network average of the inverse path lengths between any two nodes [19]. For social networks with low global efficiency, the average distance between nodes is higher than when the global efficiency is high.
7 Transitivity [20]: This parameter measures the probability that the adjacent nodes of a node are connected. Transitivity is also called the Global Clustering Coefficient.
8 Density: This parameter measures the proportion of direct connections to the total number of possible connections [57]. A dense social network contains many highly connected nodes.
9 Weighted Total Interaction: This parameter is the total number of weighted interactions between all nodes. If the weight is neglected, one obtains the Unweighted Total Interaction.

### Average network construction

In order to represent several experimental repeats as a single network with the same features as the individual networks, a methodology is proposed to construct a single graph from a set of multiple graphs. If D = (X_1_, …, X_k_) is a sample of k graphs, then the standard method of calculating the mean graph, $M\;=\;\frac{1}{k}(\sum_{i\;=\;1}^{k}X_{i})$ will fail because it assumes that the fly representing a particular node is the same for all graphs. However, in our experiments, each graph is constructed using a new set of flies. A correspondence between the flies in the different graphs should be established. Such a correspondence is established by an optimization-based graph matching technique [58,59]. If W_1_ and W_2_ represent 2 (*m* × *m*) networks, then both graphs are matched by minimizing the Frobenius norm ∥*W*_1_ –*PW*_2_*P*^*T*^∥, where P is the set of all (*m* × *m*) permutation matrices [33]. Since this minimization problem is NP-hard, we use the Graduated Assignment algorithm [60]. Once all the graphs are matched, there is a correspondence between the flies in one network to the flies in the other networks., i.e. fly 1 representing node 1 of graph 1 corresponds to fly 1 representing node 1 of graph 2 and so on. Then, the standard method can be used to compute the average network $M\;=\;\frac{1}{k}(\sum_{i\;=\;1}^{k}{X\prime }_{i})$, where *X*′_*i*_ represents the matched network of the *i*^th^ experiment.

Algorithm 1: Algorithm to compute the average network of a set of k social interaction networks. Input: a set of k (m x m) interaction matrices each representing a social interaction network. Output: an average network of the set of k originals networks.

Step 1: Choose one interaction matrix W_1_ and set the mean matrix M = W_1_

Step 2: For i = 2 to k

1. Choose interaction matrix W_i_ and compute permutation matrix R which minimizes the Frobenius norm ∥*M* − *R W*_*i*_ *R*^*T*^∥. Reorder the nodes of W_i_ by setting $W_{i}^{o}\;=\;R^{T}W_{i}R$
2. Update the mean matrix, $M\;=\;\frac{i-1}{i}M+\frac{1}{i}W_{i}^{o}$

Step 3: End

### RNA sequencing

Isolated or control flies were collected on the day of experiment. Isolation or grouped housing lasted for six days. Three repeats of each experiment were performed, with 16 flies in each group. Flies were collected 2–4 hours after the light cycle starts. Cold anesthesia was used to immobile the flies and to transfer them into RNA free tubes with beads. Following the TRIzol (Invitrogen) protocol [55]. In short, 16 whole bodies of fruit flies were homogenized and RNA was extracted after phase preparation and precipitation. RNA sequencing was done following standard protocols of Illumina NextSeq.

### RNA sequencing analysis

Raw sequence reads from the FASTQ files of 24 samples were mapped against the *Drosophila melanogaster* reference genome dm3 using STAR2.3.1t with default parameters [61]. Only the uniquely mapped reads were used in the following analysis. To identify the differentially expressed genes, the reads were counted against the Ensembl *Drosophila melanogaster* annotation BDPG5 to calculate the numbers of reads per gene. The counts of all samples were tabulated. Then this table was fed to DESeq [62] for normalization and identification of differentially expressed genes between control and isolated groups for all four time points (1 day, 2 day, 3 day and 6 day) using the standard workflow as previously described [62–64]. The Benjamini-Hochberg procedure [65] was used to correct for multiple hypothesis testing. The differentially expressed genes are identified with an FDR cutoff of 0.05.

To identify the differentially alternatively spliced events, the reads were count against the gene models generated from the Ensembl *Drosophila melanogaster* annotation BDPG5 to calculate the number of reads on the splicing junctions. The junction counts were then processed to extract the splicing events. The counts for each event was tabulated and the differential splicing of each event between control and isolated groups for all four time points (1 day, 2 day, 3 day and 6 day) was tested using the Dirichlet multinomial model as previously described [37]. The Benjamini-Hochberg procedure [65] was used to correct for multiple hypothesis testing. The differentially alternative spliced events were identified with an FDR cutoff of 0.05. Gene Ontology (GO) analysis for differentially expressed genes of all comparisons was performed using Fisher’s exact test.

### Weight and lipid measurements

The dry weights of adult flies were measured with a Mettler-Toledo XS105 precision balance in groups of 16 from isolated or grouped housed flies (see the isolation part of the Methods for details). After dry weight measurements, the adults were put in the same groups into Falcon tubes, which were racked overnight in an oven at 50°C containing silica gel. The next day the dry weight of the flies was measured. The lipid content of the same groups of flies was measured by lipid extraction using petroleum ether (Sigma-Aldrich, 320447, adapted from Fairbanks and Burch [66]). To measure lipid content, the same groups of flies were placed overnight in sealed test tubes containing 3 mL of petroleum ether. The next day the ether was poured away and the larvae were dried for several hours at 50°C. The total lipid content was calculated as the dry weight subtracted from the dry weight after ether extraction. The percentage of lipid content from the dry weight was calculated afterwards.

### Statistical analysis

Since the data was not normal distributed, we used non-parametric statistical analysis for locomotion activity and social activity. Walking distance is analyzed using unpaired t test with Welch’s correction for isolation experiment and two-way ANOVA with multiple comparisons within control or isolation groups over 6 days. In isolation experiments, we used the non-parametric Mann-Whitney test to compare social parameters between isolation group and control group. Non-parametric two-way ANOVA (Scheirer-Ray-Hare extension to Kruskal-Wallis) test was used for post-hoc multiple comparison in experiments where flies were isolated for different days. Fisher’s exact test was used for GO enrichment analysis. D’Agostino & Pearson normality test was used for normality test of lipid/dry-weight analysis. Paired t-test was used for comparison between control and isolation groups in lipid weight experiment.

## Supporting information

S1 Fig Describes a bipartite graph for splitting connected components containing interacting flies. The nodes P1,.., P4 represent the individual flies in the previous frame while C1 and C2 represent the candidate connected components containing multiple interacting flies. C1_1_, C2_1_ are duplicated nodes. After solving the assignment problem, a minimum weighted graph is obtained where the nodes P1 and P2 are connected to C1 while P3 and P4 are connected to C2. Each of the 2 connected components thus contains 2 individual flies.
(TIF)

S2 Fig Original social networks and corresponding average network. On the left are the different networks constructed from 10 biological repeats of an experiment under the same conditions. Each network was constructed from its corresponding adjacency matrix, normalized to the maximum number of interactions within that matrix. On the right is the average network, reconstructed based on the graph matching method.
(TIF)

S3 Fig Social parameters calculated from the isolation experiment. The individual network parameters In-Degree, Out-Degree and Degree all show significant differences with p<0.0001. (D) No significant difference between isolated and control groups was observed for the local parameter Betweenness Centrality, demonstrating that pivot flies do not significantly gain in importance. Significant differences between control and isolation groups were observed for the weighted individual parameters Weighted In-/Out-Degrees (G-H). The global parameters Density and Transitivity show no significant differences. All plots use 2.5th to 97.5th percentiles as whiskers and 25th to 75th percentiles as box, with the median value in the middle of the box (n = 10).
(TIF)

S4 Fig Evolution of social network parameters over different days of isolation. Different parameters show changes over time and differences between control (blue) and isolated (red) groups. Significance is noted with asterisks, the number of which represent the negative orders of magnitude of p value. Data are shown in median with interquartile ranges (n = 10 on each day in both groups).
(TIF)

S5 Fig Genome-wide analysis and physiological changes. (A, B) Scatter plots of log_2_ of fold changes versus log_10_ of gene counts on days 2 and 3. (C) GO analysis shows up-regulated genes on day 2 are mainly related to iron ion binding. (D) GO analysis shows down-regulated genes on day 2 are mainly clustered into carboxylase and amino metabolic processes. (E-H) Wet, dry, ether and lipid weight changes between control and isolation groups in male and female groups. (male: n = 25, female: n = 22). All p values are less than 0.05 in paired t-test.
(TIF)

S6 Fig The example network constructions. (A) The matrix consists of the number of interactions between any two flies. For example, row 1 column 2 indicates 2 interactions from fly X1 to fly X2, which is shown in (B) as an arrow from X1 to X2, whose thickness is proportional to the number of interactions.
(TIF)

S7 Fig Correlation analysis among different parameters between control and isolation flies on day 1, 2 and 6. Each filled circle represents the correlation coefficient with p value less than 0.01. The size and color of the circle are proportional to the correlation coefficients and blue colors indicate positive correlations while red colors indicate negative correlations. On Day 1 the total walking distance showed positive correlations to all individual parameters and clustering coefficient in isolation group compared to control group. For the remaining days, the correlation map is similar between control and isolation groups.
(TIF)

S1 Video Tracking results of 10 walking flies in the Flyworld arena by Flytracker. Each fly is labelled blue while trajectories are labelled with different colours. The video is a 15 minute clip of a 1-hour tracking video.
(MP4)

S2 Video Tracking results of 16 walking flies in the Flyworld arena by Flytracker. Each fly is labelled with a number and a distinct colour for its trajectory (+/- 0.5 s). The video is a 30 second clip of a 1-hour tracking video.
(MP4)

S3 Video Tracking results of 50 walking flies in the Flyworld arena by Flytracker. Each fly is labelled with a distinct colour for its trajectory (from 0.5s before current position). The video is an 8 second clip of a 1-hour tracking video.
(MP4)

S4 Video An annotated video clip from a 6-min sample video showing the tracking errors by Ctrax on 10 flies walking in the Flyworld arena. The colored shapes represent different flies that Ctrax detects. This clip started from when the errors become severe. The errors include losing identity (no color contour covers the fly), swapping identity rapidly (change of colors), emerging new false identity, and false connection (odd connection line), etc. Using the Fixerror tool provided along with Ctrax has already fixed some errors in the beginning of the original video although the errors still occur as shown in this clip.
(MOV)

S5 Video A 10-second video clip with tracking results of 10 flies by Flytracker from Buridan’s paradigm. Two female and eight male flies with clipped wings walking on the platform surrounded by water to prevent them walking outside of the platform. All flies were labeled with detected identity.
(MP4)

S6 Video A 6-second video clip for manual checking the tracking results from a 50-fly one-hour video. Each time, the observer focuses on one single fly (Fly 1 in this video in a black box with its track as black dots in frames [-30, +30]). All flies were labeled with their corresponding identity numbers to help the observer to check if there are missing or swapped identities.
(MP4)

S7 Video Classification of a touch behavior between two flies. An interactor approaches the rear part of the interacted and the interaction is considered valid only when two flies maintain relative positions for more than 15 frames (~0.5 s).
(MP4)

S1 Table Social isolation induced differentially alternative splicing events. Note. PATH1&2 A path includes the alternative exons and the flanking exons from one of the two isoforms of the event. The two paths of an event describe the two alternatively used isoforms of the splicing event. Event Types: A5SS: Alternative 5’ splicing sites; AFE: Alternative first exons; ES: Exon skipping, MC: Multi-comparison, there are more than 2 paths in comparison.
(PDF)

S2 Table Individual and local social network parameters of sample network calculated for each individual object. (PDF)

S3 Table Global parameters of sample network calculated for the whole group of objects. (PDF)

S1 File Documents of Flyworld and Buridan setup designs. In the zip file, there are 3D model designs of key parts of Flyworld used in this experiment in folder “Flyworld” and designs of Buridan’s Paradigm for testing Flytracker in the folder “Buridan setup”. All 3D designs are made using SolidWorks which can be viewed and measured using the free software “eDrawings”.
(ZIP)

## References

[1] Likitlersuang J, Stephens G, Palanski K, Ryu WS. *C*. *elegans* Tracking and Behavioral Measurement. J Vis Exp. 2012; 10.3791/4094 23183548 PMC3523420

[2] Sokolowski MB. Social Interactions in “Simple” Model Systems. Neuron. Elsevier Ltd; 2010;65: 780–794. 10.1016/j.neuron.2010.03.007 20346755

[3] Cross SE, Morris ML, Gore JS. Thinking about oneself and others: The relational-interdependent self-construal and social cognition. J Pers Soc Psychol. 2002;82: 399–418. 10.1037//0022-3514.82.3.399 11902624

[4] Macrae CN, Bodenhausen G V. Social Cognition: Thinking Categorically about Others. Annu Rev Psychol. 2000;51: 93–120. 10.1146/annurev.psych.51.1.93 10751966

[5] Simon AF, Chou MT, Salazar ED, Nicholson T, Saini N, Metchev S, et al A simple assay to study social behavior in Drosophila: Measurement of social space within a group. Genes, Brain Behav. 2012;11: 243–252. 10.1111/j.1601-183X.2011.00740.x 22010812 PMC3268943

[6] Stranahan AM, Khalil D, Gould E. Social isolation delays the positive effects of running on adult neurogenesis. Nat Neurosci. 2006;9: 526–533. 10.1038/nn1668 16531997 PMC3029943

[7] Harlow HF, Dodsworth RO, Harlow MK. Total social isolation in monkeys. Proc Natl Acad Sci U S A. 1965;54: 90–97. Available: http://www.ncbi.nlm.nih.gov/pmc/articles/PMC285801/4955132 10.1073/pnas.54.1.90 PMC285801

[8] Ruan H, Wu C-F. Social interaction-mediated lifespan extension of Drosophila Cu/Zn superoxide dismutase mutants. Proc Natl Acad Sci U S A. 2008;105: 7506–10. 10.1073/pnas.0711127105 18508973 PMC2396722

[9] Nonogaki K, Nozue K, Oka Y. Social isolation affects the development of obesity and type 2 diabetes in mice. Endocrinology. 2007;148: 4658–4666. 10.1210/en.2007-0296 17640995

[10] Karelina K, Norman GJ, Zhang N, Morris JS, Peng H, DeVries AC. Social isolation alters neuroinflammatory response to stroke. Proc Natl Acad Sci. 2009;106: 5895–5900. 10.1073/pnas.0810737106 19307557 PMC2667090

[11] Seeman TE. Social ties and health: The benefits of social integration. Ann Epidemiol. 1996;6: 442–451. 10.1016/S1047-2797(96)00095-6 8915476

[12] Powell ND, Sloan EK, Bailey MT, Arevalo JMG, Miller GE, Chen E, et al Social stress up-regulates inflammatory gene expression in the leukocyte transcriptome via -adrenergic induction of myelopoiesis. Proc Natl Acad Sci. 2013;110: 16574–16579. 10.1073/pnas.1310655110 24062448 PMC3799381

[13] Cole SW. Human Social Genomics. PLoS Genet. 2014;10 10.1371/journal.pgen.1004601 25166010 PMC4148225

[14] Cole SW, Hawkley LC, Arevalo JM, Sung CY, Rose RM, Cacioppo JT. Social regulation of gene expression in human leukocytes. Genome Biol. 2007;8 10.1186/gb-2007-8-9-r189 17854483 PMC2375027

[15] Kaidanovich-Beilin O, Lipina T, Vukobradovic I, Roder J, Woodgett JR. Assessment of social interaction behaviors. J Vis Exp. 2011;0: 6–10. 10.3791/2473 21403628 PMC3197404

[16] Krupp JJ, Kent C, Billeter JC, Azanchi R, So AKC, Schonfeld JA, et al Social Experience Modifies Pheromone Expression and Mating Behavior in Male Drosophila melanogaster. Curr Biol. 2008;18: 1373–1383. 10.1016/j.cub.2008.07.089 18789691

[17] Dankert H, Wang L, Hoopfer ED, Anderson DJ, Perona P. Automated monitoring and analysis of social behavior in Drosophila. Nat Methods. 2009;6: 297–303. 10.1038/nmeth.1310 19270697 PMC2679418

[18] Nagy M, Ákos Z, Biro D, Vicsek T. Hierarchical group dynamics in pigeon flocks. Nature. 2010;464: 890–893. 10.1038/nature08891 20376149

[19] Latora V, Marchiori M. Efficient behavior of small-world networks. Phys Rev Lett. 2001;87: 198701-1–198701–4. 10.1103/PhysRevLett.87.198701 11690461

[20] Schneider J, Dickinson MH, Levine JD. Social structures depend on innate determinants and chemosensory processing in Drosophila. Proc Natl Acad Sci. 2012;109: 17174–17179. 10.1073/pnas.1121252109 22802679 PMC3477376

[21] Wey T, Blumstein DT, Shen W, Jordán F. Social network analysis of animal behaviour: a promising tool for the study of sociality. Anim Behav. 2008;75: 333–344. 10.1016/j.anbehav.2007.06.020

[22] Makagon MM, McCowan B, Mench JA. How can social network analysis contribute to social behavior research in applied ethology? Appl Anim Behav Sci. Elsevier B.V.; 2012;138: 152–161. 10.1016/j.applanim.2012.02.003 24357888 PMC3865988

[23] Branson K, Robie AA, Bender J, Perona P, Dickinson MH. High-throughput ethomics in large groups of Drosophila. Nat Methods. Nature Publishing Group; 2009;6: 451–457. 10.1038/nmeth.1328 19412169 PMC2734963

[24] Chao R, Macía-Vázquez G, Zalama E, Gómez-García JB, Perán JR. Automated tracking of drosophila specimens. Sensors (Switzerland). 2015;15: 19369–19392. 10.3390/s150819369 26258779 PMC4570375

[25] Ardekani R, Biyani A, Dalton JE, Saltz JB, Arbeitman MN, Tower J, et al Three-dimensional tracking and behaviour monitoring of multiple fruit flies. J R Soc Interface. 2012;10: 20120547–20120547. 10.1098/rsif.2012.0547 23034355 PMC3565780

[26] Pérez-Escudero A, Vicente-Page J, Hinz RC, Arganda S, De Polavieja GG. IdTracker: Tracking individuals in a group by automatic identification of unmarked animals. Nat Methods. 2014;11: 743–748. 10.1038/nmeth.2994 24880877

[27] Schneider J, Levine JD. Automated identification of social interaction criteria in Drosophila melanogaster. Biol Lett. 2014;10: 20140749–20140749. 10.1098/rsbl.2014.0749 25354920 PMC4272216

[28] Kabra M, Robie AA, Rivera-Alba M, Branson S, Branson K. JAABA: Interactive machine learning for automatic annotation of animal behavior. Nat Methods. 2013;10: 64–67. 10.1038/nmeth.2281 23202433

[29] Simon JC, Dickinson MH. A new chamber for studying the behavior of Drosophila. PLoS One. 2010;5 10.1371/journal.pone.0008793 20111707 PMC2811731

[30] Nath T, Liu G, Weyn B, Hassan B, Ramaekers A, De Backer S, et al. Tracking for quantifying social network of Drosophila melanogaster. Lecture Notes in Computer Science (including subseries Lecture Notes in Artificial Intelligence and Lecture Notes in Bioinformatics). 2013. pp. 539–545. 10.1007/978-3-642-40246-3_67

[31] Colomb J, Reiter L, Blaszkiewicz J, Wessnitzer J, Brembs B. Open source tracking and analysis of adult Drosophila locomotion in Buridan’s paradigm with and without visual targets. PLoS One. 2012;7 10.1371/journal.pone.0042247 22912692 PMC3415391

[32] Nath T, Liu G, Hassan B, Weyn B, De Backer S, Scheunders P. Automated social behaviour recognition at low resolution. Proc—Int Conf Pattern Recognit. 2014; 2323–2328. 10.1109/ICPR.2014.403

[33] Jain BJ, Obermayer K. Algorithms for the Sample Mean of Graphs BT—Computer Analysis of Images and Patterns. In: Jiang X, Petkov N, editors. Berlin, Heidelberg: Springer Berlin Heidelberg; 2009 pp. 351–359.

[34] Robinson GE, Fernald RD, Clayton DF. Genes and social behavior. Science. 2008 pp. 896–900. 10.1126/science.1159277 18988841 PMC3052688

[35] Huang DW, Sherman BT, Lempicki RA. Systematic and integrative analysis of large gene lists using DAVID bioinformatics resources. Nat Protoc. 2009;4: 44–57. 10.1038/nprot.2008.211 19131956

[36] Huang DW, Sherman BT, Lempicki RA. Bioinformatics enrichment tools: Paths toward the comprehensive functional analysis of large gene lists. Nucleic Acids Res. 2009;37: 1–13. 10.1093/nar/gkn923 19033363 PMC2615629

[37] Yu P, Shaw CA. An efficient algorithm for accurate computation of the Dirichlet- multinomial log-likelihood function. Bioinformatics. 2014;30: 1547–1554. 10.1093/bioinformatics/btu079 24519380 PMC4081639

[38] Qiu F. Myofilin, a protein in the thick filaments of insect muscle. J Cell Sci. 2005;118: 1527–1536. 10.1242/jcs.02281 15769842

[39] Rascle A, Stowers RS, Garza D, Lepesant JA, Hogness DS. L63, the Drosophila PFTAIRE, interacts with two novel proteins unrelated to cyclins. Mech Dev. 2003;120: 617–628. 10.1016/S0925-4773(03)00019-4 12782278

[40] Consortium FB. Flybase—the Drosophila database. Nucleic Acids Res. 1994;22: 3456–3458. 10.1093/nar/22.17.3456 7937045 PMC308301

[41] Johnstone O, Lasko P. Translational regulation and RNA localization in Drosophila oocytes and embryos. Annu Rev Genet. 2001;35: 365–406. 10.1146/annurev.genet.35.102401.090756 11700288

[42] Ueyama M, Chertemps T, Labeur C, Wicker-Thomas C. Mutations in the desat1 gene reduces the production of courtship stimulatory pheromones through a marked effect on fatty acids in Drosophila melanogaster. Insect Biochem Mol Biol. England; 2005;35: 911–920. 10.1016/j.ibmb.2005.03.007 15944086

[43] Wicker-Thomas C, Henriet C, Dallerac R. Partial characterization of a fatty acid desaturase gene in Drosophila melanogaster. Insect Biochem Mol Biol. 1997;27: 963–972. 10.1016/s0965-1748(97)00077-5 9501419

[44] Houot B, Bousquet F, Ferveur JF. The consequences of regulation of desat1 expression for pheromone emission and detection in Drosophila melanogaster. Genetics. 2010;185: 1297–1309. 10.1534/genetics.110.117226 20516499 PMC2927757

[45] Freeman WJ. A field-theoretic approach to understanding scale-free neocortical dynamics. Biol Cybern. 2005;92: 350–359. 10.1007/s00422-005-0563-1 15900484

[46] Newman MEJ. Scientific collaboration networks. I. Network construction and fundamental results. Phys Rev E. 2001;64: 16131 10.1103/PhysRevE.64.016131 11461355

[47] Milo R, Shen-Orr S, Itzkovitz S, Kashtan N, Chklovskii D, Alon U. Network motifs: Simple building blocks of complex networks. Science (80-). 2002;298: 824–827. 10.1126/science.298.5594.824 12399590

[48] Cacioppo JT, Hawkley LC. Perceived social isolation and cognition. Trends in Cognitive Sciences. 2009 pp. 447–454. 10.1016/j.tics.2009.06.005 19726219 PMC2752489

[49] Kumari A, Singh P, Baghel MS, Thakur MK. Social isolation mediated anxiety like behavior is associated with enhanced expression and regulation of BDNF in the female mouse brain. Physiol Behav. 2016;158: 34–42. 10.1016/j.physbeh.2016.02.032 26921097

[50] Padfield D, Rittscher J, Roysam B. Coupled minimum-cost flow cell tracking for high-throughput quantitative analysis. Med Image Anal. 2011;15: 650–668. 10.1016/j.media.2010.07.006 20864383

[51] Salvatore J. Bipartite graphs and problem solving. Univ Chicago 2007;

[52] KH W. The Hungarian method for the assignment problem. Nav Res Logist Q. Wiley-Blackwell; 1955;2: 83–97. 10.1002/nav.3800020109

[53] Munkres J. Algorithms for the Assignment and Transportation Problems. J Soc Ind Appl Math. 1957;5: 32–38. 10.1137/0105003

[54] Fagiolo G. Clustering in complex directed networks. Phys Rev E—Stat Nonlinear, Soft Matter Phys. 2007;76 10.1103/PhysRevE.76.026107 17930104

[55] Rubinov M, Sporns O. Complex network measures of brain connectivity: Uses and interpretations. Neuroimage. 2010;52: 1059–1069. 10.1016/j.neuroimage.2009.10.003 19819337

[56] Brandes U. A faster algorithm for betweenness centrality. J Math Sociol. 2001;25: 163–177. 10.1080/0022250X.2001.9990249

[57] Hanneman R a., Riddle M. Introduction to social network methods. Network. 2005; 149.

[58] Jiang X, Münger A, Bunke H. On median graphs: Properties, algorithms, and applications. IEEE Trans Pattern Anal Mach Intell. 2001;23: 1144–1151.

[59] Jain B, Obermayer K. On the sample mean of graphs. 2008; 993–1000. 10.1109/IJCNN.2008.4633920

[60] Gold S, Rangarajan A. A graduated assignment algorithm for graph matching. IEEE Trans Pattern Anal Mach Intell. 1996;18: 377–388. 10.1109/34.491619

[61] Dobin A, Davis CA, Schlesinger F, Drenkow J, Zaleski C, Jha S, et al STAR: Ultrafast universal RNA-seq aligner. Bioinformatics. 2013;29: 15–21. 10.1093/bioinformatics/bts635 23104886 PMC3530905

[62] Anders S, Huber W. Differential analysis of RNA-Seq data at the gene level using the DESeq package. 2016; 1–32. https://bioconductor.org/packages/3.3/bioc/vignettes/DESeq/inst/doc/DESeq.pdf

[63] Guo Z, González JF, Hernandez JN, McNeilly TN, Corripio-Miyar Y, Frew D, et al Possible mechanisms of host resistance to Haemonchus contortus infection in sheep breeds native to the Canary Islands. Sci Rep. 2016;6 10.1038/srep26200 27197554 PMC4873755

[64] Qian X, Li X, Ilori TO, Klein JD, Hughey RP, Li C jun, et al RNA-seq analysis of glycosylation related gene expression in STZ-induced diabetic rat kidney inner medulla. Front Physiol. 2015;6 10.3389/fphys.2015.00274 26483702 PMC4590316

[65] Benjamini Y, Hochberg Y. Controlling the false discovery rate: a practical and powerful approach to multiple testing [Internet]. Journal of the Royal Statistical Society B. 1995 pp. 289–300.

[66] Fairbanks LD, Burch GE. Rate of water loss and water and fat content of adult Drosophila melanogaster of different ages. J Insect Physiol. England; 1970;16: 1429–1436. 10.1016/0022-1910(70)90141-1 5432386

