# Supplementary figures and images for: A simple computer vision pipeline reveals the effects of isolation on social interaction dynamics in Drosophila
Source: PLoS Comput Biol. 2018 Aug 30;14(8):e1006410. doi: 10.1371/journal.pcbi.1006410 (PMC6135522; doi:10.1371/journal.pcbi.1006410)

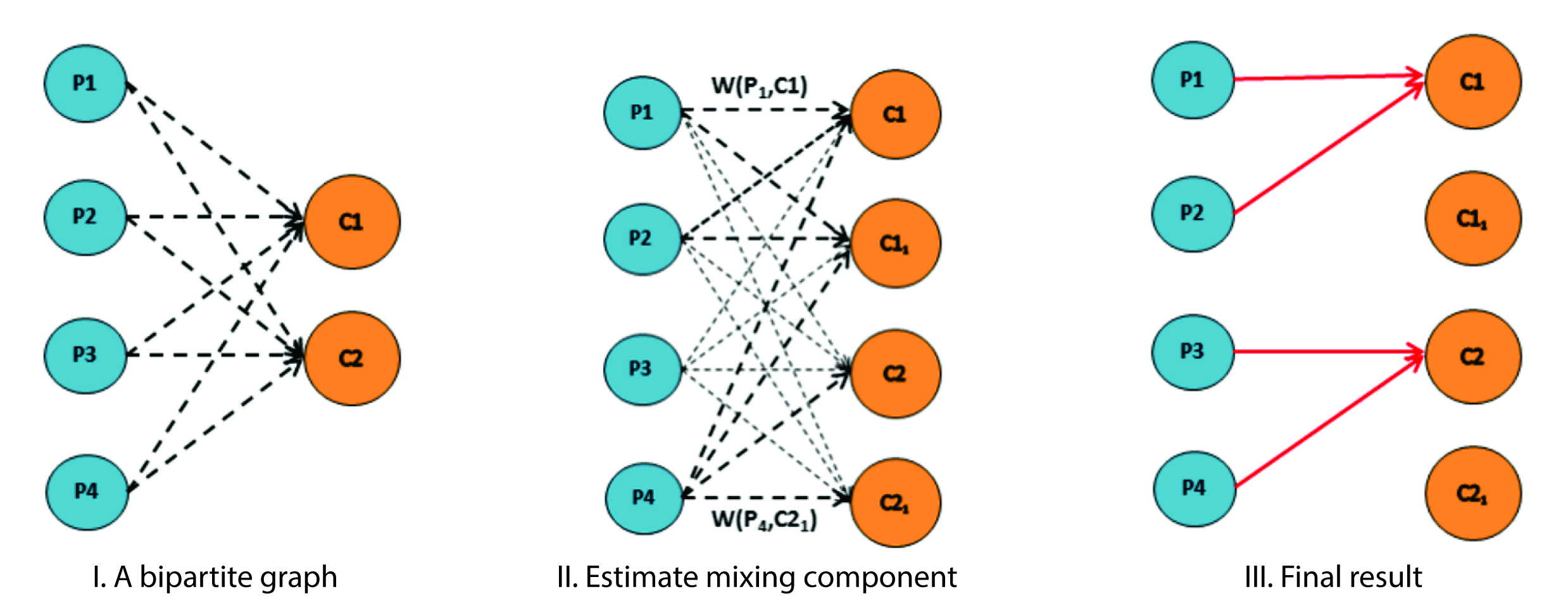

Supplement: S1 Fig — The nodes P1,.., P4 represent the individual flies in the previous frame while C1 and C2 represent the candidate connected components containing multiple interacting flies. C11, C21 are duplicated nodes. After solving the assignment problem, a minimum weighted graph is obtained where the nodes P1 and P2 are connected to C1 while P3 and P4 are connected to C2. Each of the 2 connected components thus contains 2 individual flies. (TIF) [file pcbi.1006410.s001.tif]

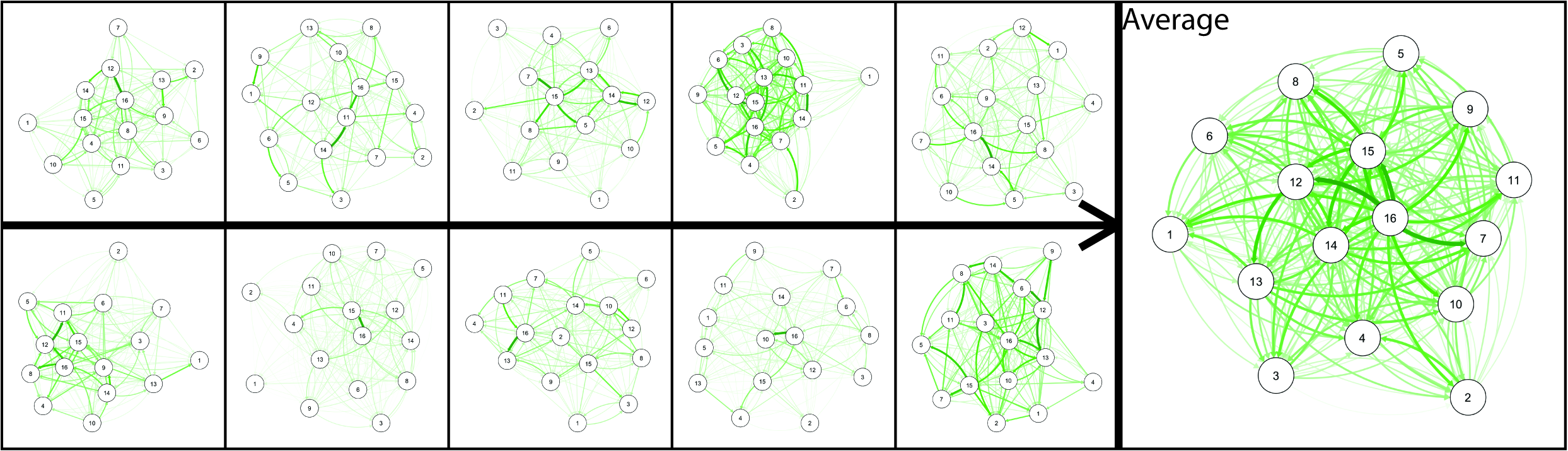

Supplement: S2 Fig — On the left are the different networks constructed from 10 biological repeats of an experiment under the same conditions. Each network was constructed from its corresponding adjacency matrix, normalized to the maximum number of interactions within that matrix. On the right is the average network, reconstructed based on the graph matching method. (TIF) [file pcbi.1006410.s002.tif]

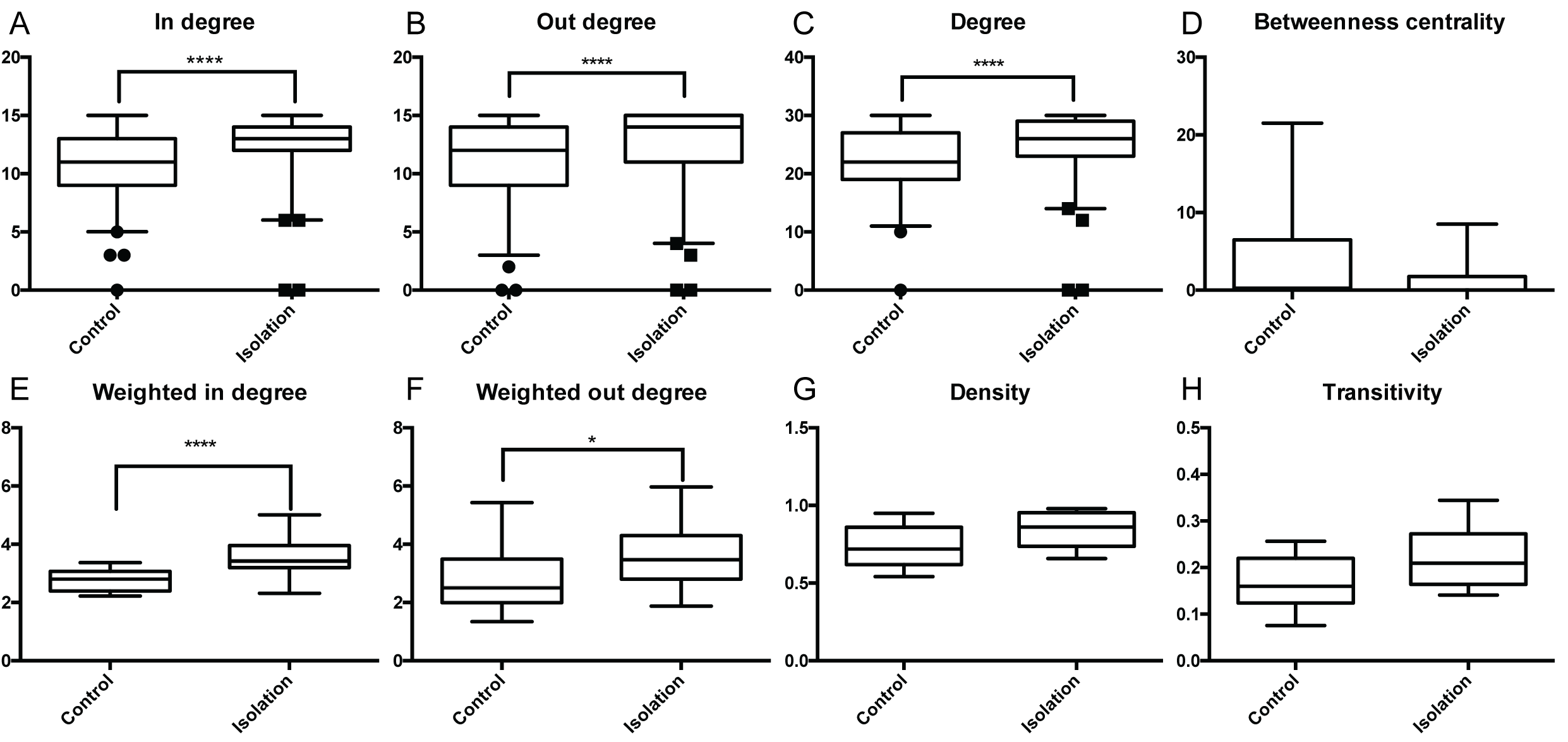

Supplement: S3 Fig — The individual network parameters In-Degree, Out-Degree and Degree all show significant differences with p<0.0001. (D) No significant difference between isolated and control groups was observed for the local parameter Betweenness Centrality, demonstrating that pivot flies do not significantly gain in importance. Significant differences between control and isolation groups were observed for the weighted individual parameters Weighted In-/Out-Degrees (G-H). The global parameters Density and Transitivity show no significant differences. All plots use 2.5th to 97.5th percentiles as whiskers and 25th to 75th percentiles as box, with the median value in the middle of the box (n = 10). (TIF) [file pcbi.1006410.s003.tif]

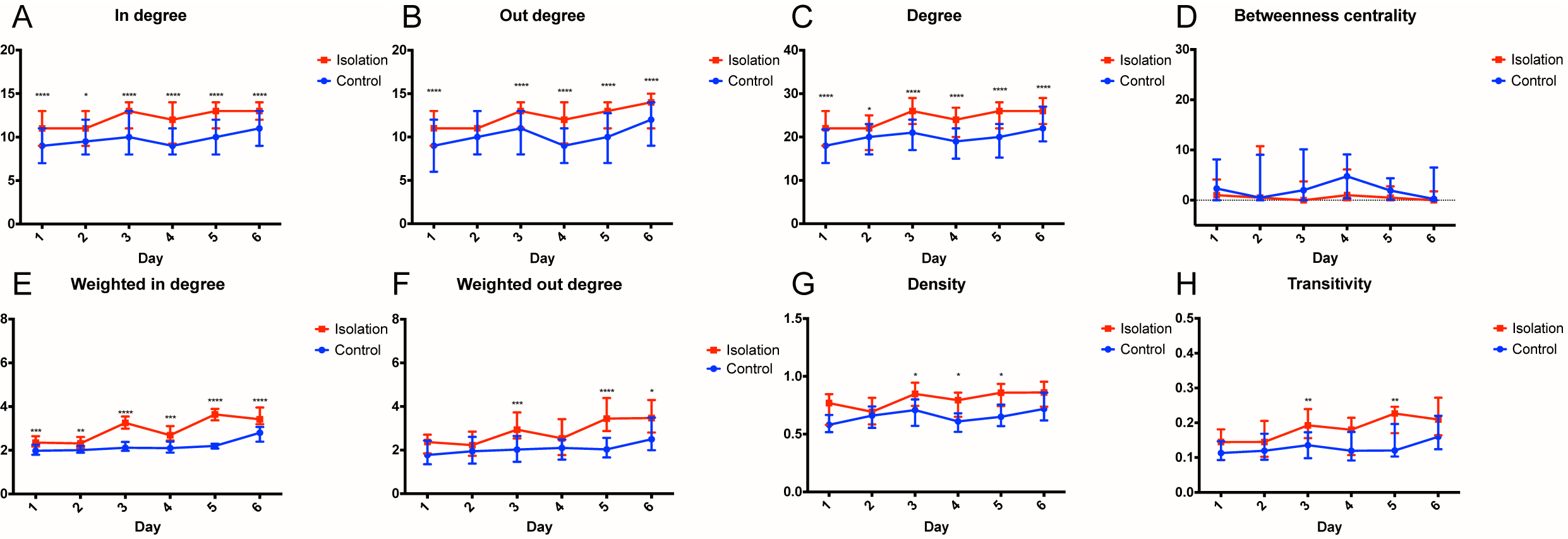

Supplement: S4 Fig — Different parameters show changes over time and differences between control (blue) and isolated (red) groups. Significance is noted with asterisks, the number of which represent the negative orders of magnitude of p value. Data are shown in median with interquartile ranges (n = 10 on each day in both groups). (TIF) [file pcbi.1006410.s004.tif]

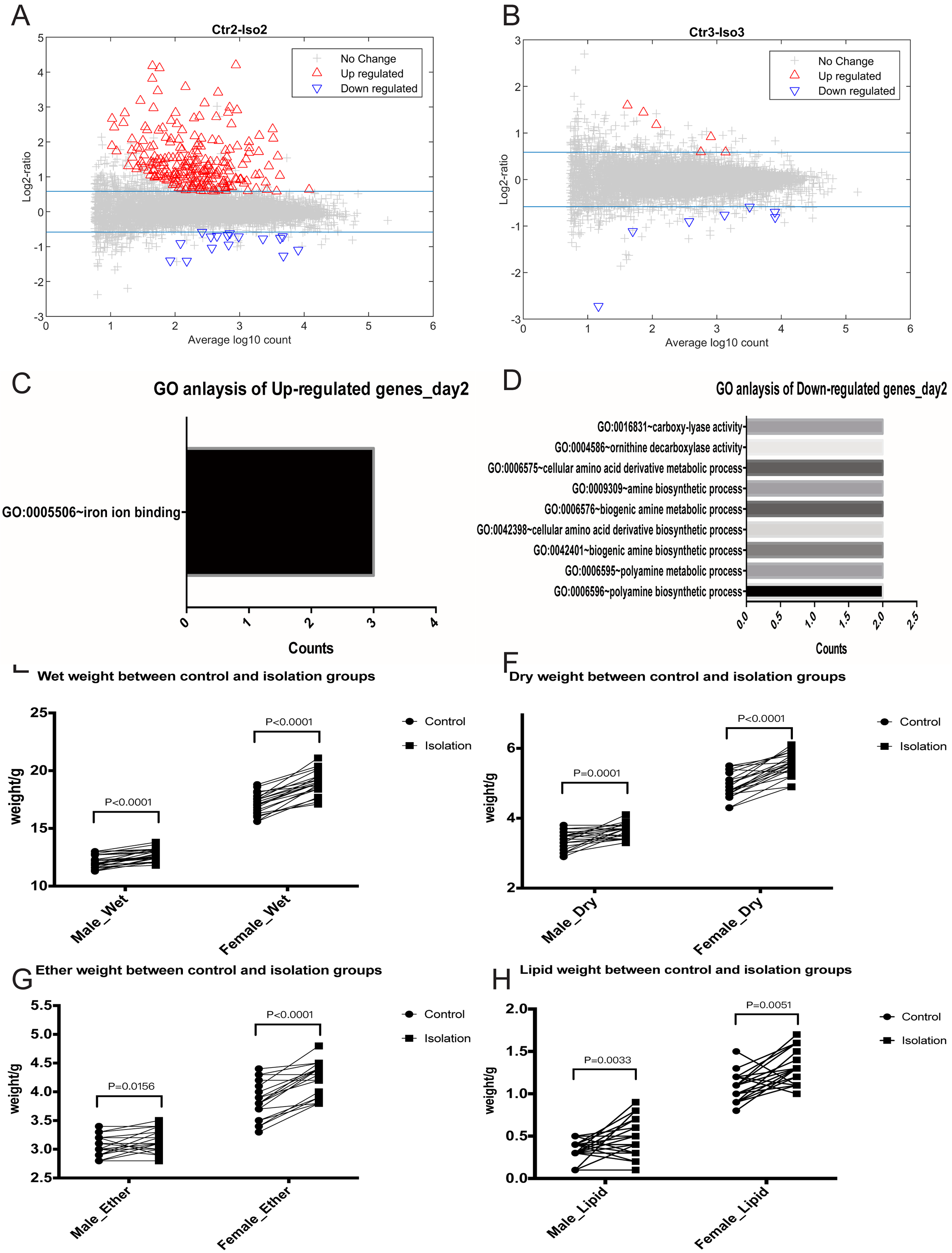

Supplement: S5 Fig — (A, B) Scatter plots of log2 of fold changes versus log10 of gene counts on days 2 and 3. (C) GO analysis shows up-regulated genes on day 2 are mainly related to iron ion binding. (D) GO analysis shows down-regulated genes on day 2 are mainly clustered into carboxylase and amino metabolic processes. (E-H) Wet, dry, ether and lipid weight changes between control and isolation groups in male and female groups. (male: n = 25, female: n = 22). All p values are less than 0.05 in paired t-test. (TIF) [file pcbi.1006410.s005.tif]

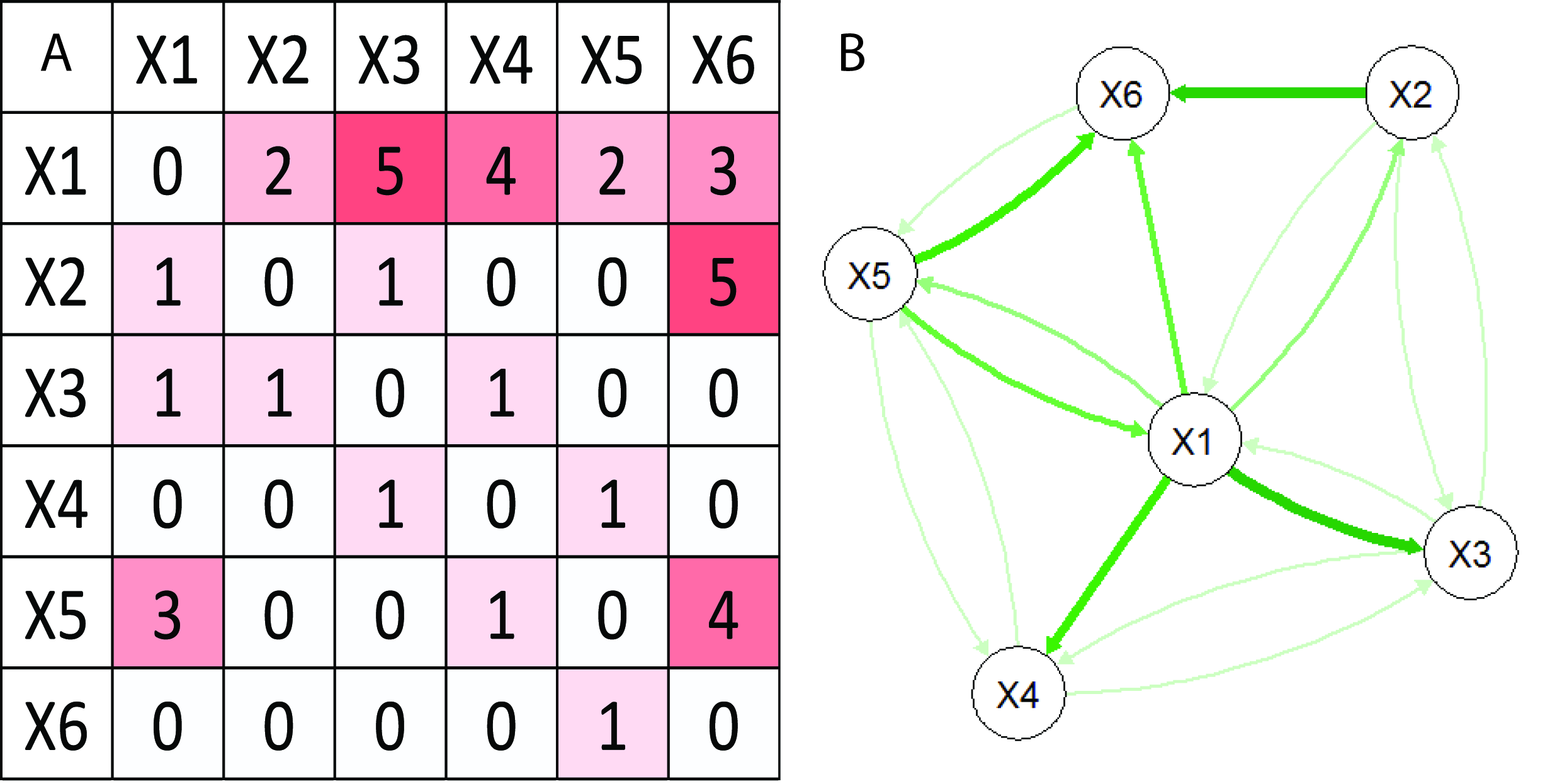

Supplement: S6 Fig — (A) The matrix consists of the number of interactions between any two flies. For example, row 1 column 2 indicates 2 interactions from fly X1 to fly X2, which is shown in (B) as an arrow from X1 to X2, whose thickness is proportional to the number of interactions. (TIF) [file pcbi.1006410.s006.tif]

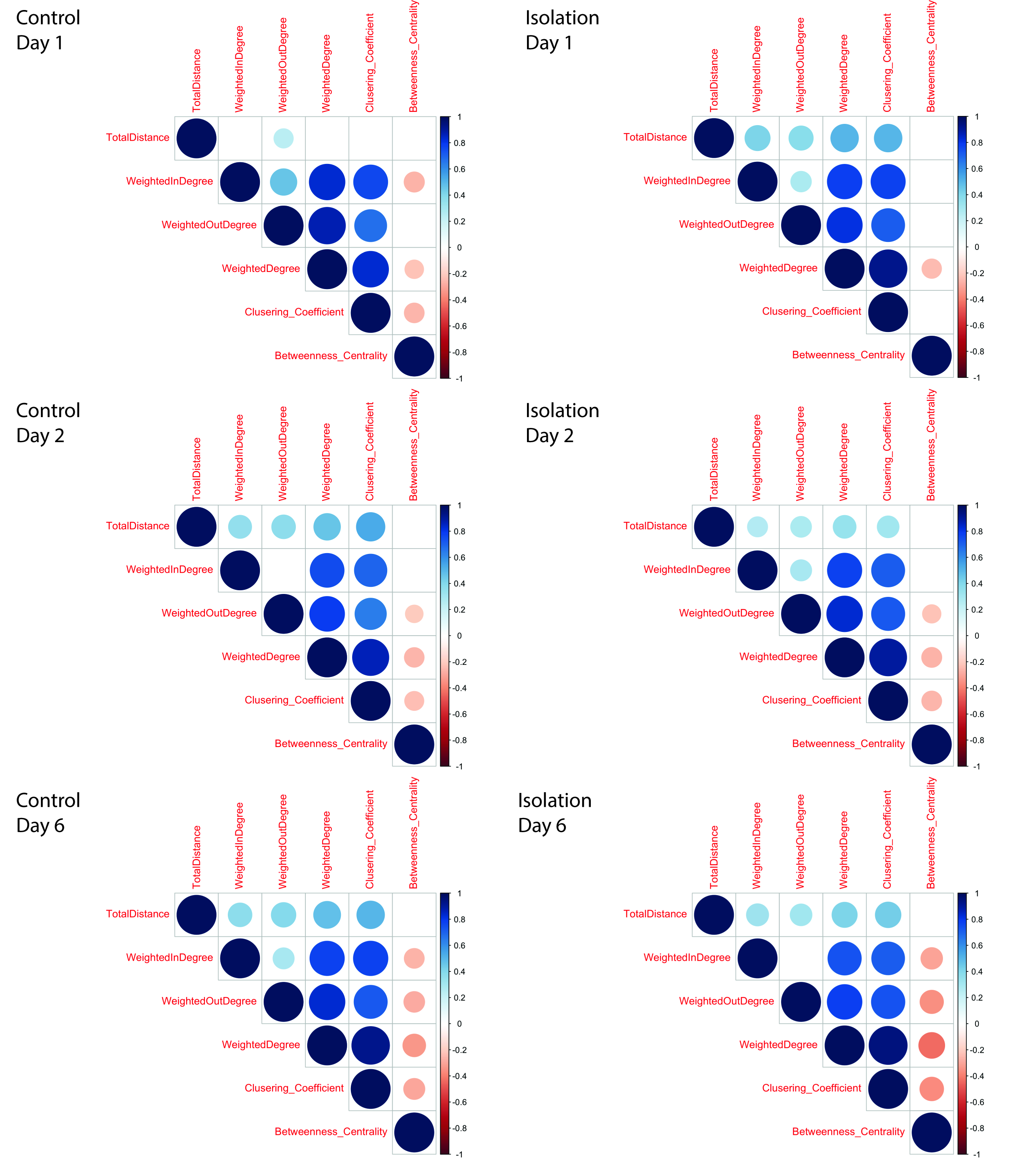

Supplement: S7 Fig — Each filled circle represents the correlation coefficient with p value less than 0.01. The size and color of the circle are proportional to the correlation coefficients and blue colors indicate positive correlations while red colors indicate negative correlations. On Day 1 the total walking distance showed positive correlations to all individual parameters and clustering coefficient in isolation group compared to control group. For the remaining days, the correlation map is similar between control and isolation groups. (TIF) [file pcbi.1006410.s007.tif]

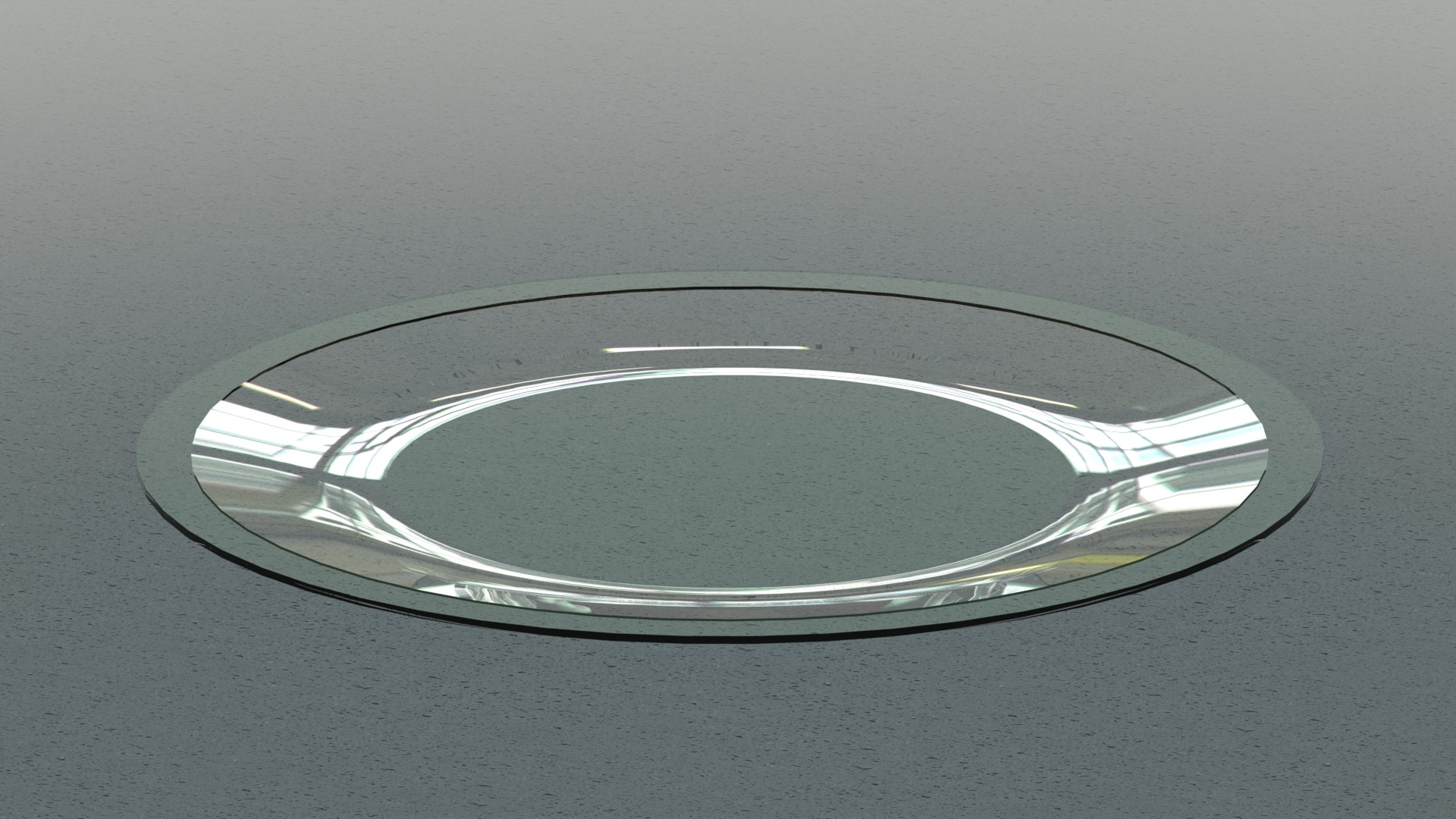

Supplement: S1 File — In the zip file, there are 3D model designs of key parts of Flyworld used in this experiment in folder “Flyworld” and designs of Buridan’s Paradigm for testing Flytracker in the folder “Buridan setup”. All 3D designs are made using SolidWorks which can be viewed and measured using the free software “eDrawings”. (ZIP) [file pcbi.1006410.s018.zip › Rendering of ArenaBottomShape.JPG]
