# Supplementary material for: A simple computer vision pipeline reveals the effects of isolation on social interaction dynamics in Drosophila
Source: PLoS Comput Biol. 2018 Aug 30;14(8):e1006410. doi: 10.1371/journal.pcbi.1006410 (PMC6135522; doi:10.1371/journal.pcbi.1006410)
Supplement: S1 Table — Note. PATH1&2 A path includes the alternative exons and the flanking exons from one of the two isoforms of the event. The two paths of an event describe the two alternatively used isoforms of the splicing event. Event Types: A5SS: Alternative 5’ splicing sites; AFE: Alternative first exons; ES: Exon skipping, MC: Multi-comparison, there are more than 2 paths in comparison. (PDF) [file pcbi.1006410.s015.pdf]

Supplementary Table 1 Social isolation induced differentially alternative splicing events.

DAYS OF ISOLATION	GENE	EVENT TYPE	PATH1	PATH2
1	Mf	A5SS, decrease	E6,E8	E6,E7,E8
2	Mf	A5SS, decrease	E6,E8	E6,E7,E8
	Pif1A,Pif1B	AFE, increase	E13,E14	E6,E9,E10,E11,E12,E14
3	CG16758	AFE, increase	E1,E5	E2,E3,E5
	sqd	ES, increase	E3,E5	E3,E4,E5
6	desat1	MC, decrease	E1,E6;E2,E6	E5,E6;E5,E6

Note. PATH1&2 A path includes the alternative exons and the flanking exons from one of the two isoforms of the event. The two paths of an event describe the two alternatively used isoforms of the splicing event. Event Types: A5SS: Alternative 5' splicing sites; AFE: Alternative first exons; ES: Exon skipping, MC: Multi-comparison, there are more than 2 paths in comparison.
