# Supplementary material for: A simple computer vision pipeline reveals the effects of isolation on social interaction dynamics in Drosophila
Source: PLoS Comput Biol. 2018 Aug 30;14(8):e1006410. doi: 10.1371/journal.pcbi.1006410 (PMC6135522; doi:10.1371/journal.pcbi.1006410)
Supplement: S2 Table — (PDF) [file pcbi.1006410.s016.pdf]

Supplementary Table 2 Individual and local social network parameters of sample network

Fly	In Degree	Out Degree	Degree	Weighted In Degree	Weighted Out Degree	Weighted Degree	Clustering Coefficient	Betweenness Centrality
**1**	3	5	8	1	3.2	4.2	0.1804	3
**2**	2	3	5	0.6	1.4	2.0	0.2228	1
**3**	3	3	6	1.4	0.6	2.0	0.1738	6.5
**4**	3	2	5	1.2	0.4	1.6	0.2148	6.0
**5**	3	3	6	0.8	1.6	2.4	0.1555	5.5
**6**	3	1	4	2.4	0.2	2.6	0.3131	0
