# Supplementary material for: A simple computer vision pipeline reveals the effects of isolation on social interaction dynamics in Drosophila
Source: PLoS Comput Biol. 2018 Aug 30;14(8):e1006410. doi: 10.1371/journal.pcbi.1006410 (PMC6135522; doi:10.1371/journal.pcbi.1006410)
Supplement: S3 Table — (PDF) [file pcbi.1006410.s017.pdf]

Supplementary Table 3 Global parameters of sample network

Global Parameters	Assortativity	Global Efficiency	Density	Transitivity	Weighted Total Interaction
Values	0.1011	0.3240	0.5667	0.1932	7.4
