# Supplementary material for: A simple computer vision pipeline reveals the effects of isolation on social interaction dynamics in Drosophila
Source: PLoS Comput Biol. 2018 Aug 30;14(8):e1006410. doi: 10.1371/journal.pcbi.1006410 (PMC6135522; doi:10.1371/journal.pcbi.1006410)
Supplement: S1 File — In the zip file, there are 3D model designs of key parts of Flyworld used in this experiment in folder “Flyworld” and designs of Buridan’s Paradigm for testing Flytracker in the folder “Buridan setup”. All 3D designs are made using SolidWorks which can be viewed and measured using the free software “eDrawings”. (ZIP) [file pcbi.1006410.s018.zip › Arena.PDF]

Revision table			
REV.	Discription	Date	Name
01	Original	24/11/2011	Manu

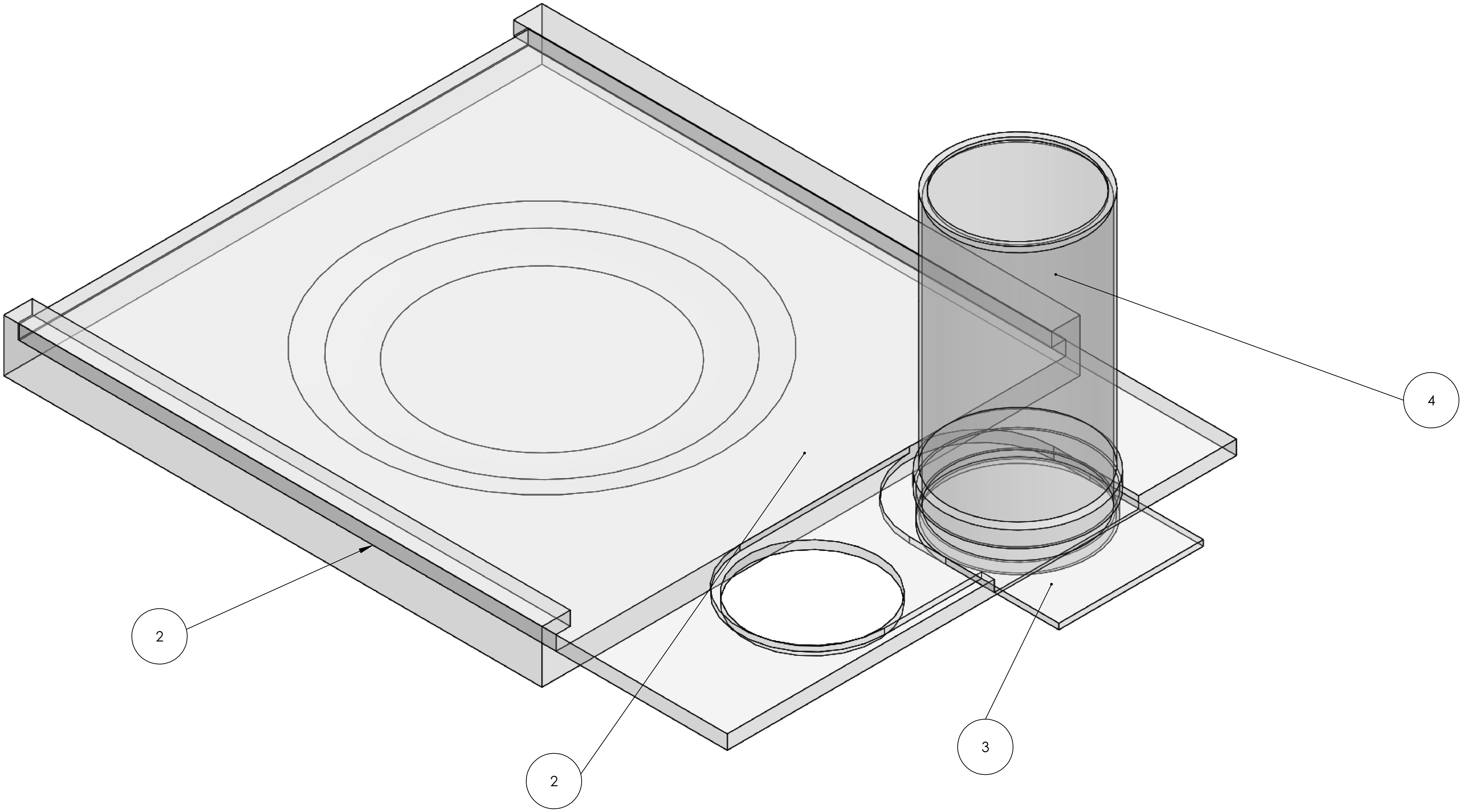

These drawings are and remain the property of Peira BVBA. Reuse or modification of the original drawings without written permission is prohibited. All rights reserved Peira BVBA.

ITEM NO.	PART NUMBER	DESCRIPTION	QTY.
1	PRA-09.006.601	Formule Vorm	1
2	PRA-09.006.602	Deksel Groot	1
3	PRA-09.006.603	Deksel	1
4	potje	Vliegenpot	1

Rough Dimentions	QTY	Material			Drawn by		Scale
L : H : W :	1				Manu		1:2
General tolerances: Length: Corner:		Surface treatment	Hardness	Finishing	Created on 24/11/2011	Weight 632.20 g	Sheetformat A3

PEIRA BVBA
Ketelaarstraat 8
2340 Beerse
www.peira.be
info@peira.be
Tel : +3214600800

Description: Spatial Fly World

Drawingnumber: PRA-09.006.600

Page: Blad 1
