# Supplementary material for: A simple computer vision pipeline reveals the effects of isolation on social interaction dynamics in Drosophila
Source: PLoS Comput Biol. 2018 Aug 30;14(8):e1006410. doi: 10.1371/journal.pcbi.1006410 (PMC6135522; doi:10.1371/journal.pcbi.1006410)
Supplement: S1 File — In the zip file, there are 3D model designs of key parts of Flyworld used in this experiment in folder “Flyworld” and designs of Buridan’s Paradigm for testing Flytracker in the folder “Buridan setup”. All 3D designs are made using SolidWorks which can be viewed and measured using the free software “eDrawings”. (ZIP) [file pcbi.1006410.s018.zip › Buridan plate.PDF]

Revision table			
REV.	Discription	Date	Name
01	Original	11/09/2014	Manus
02	Change Position	29/10/2014	Manus

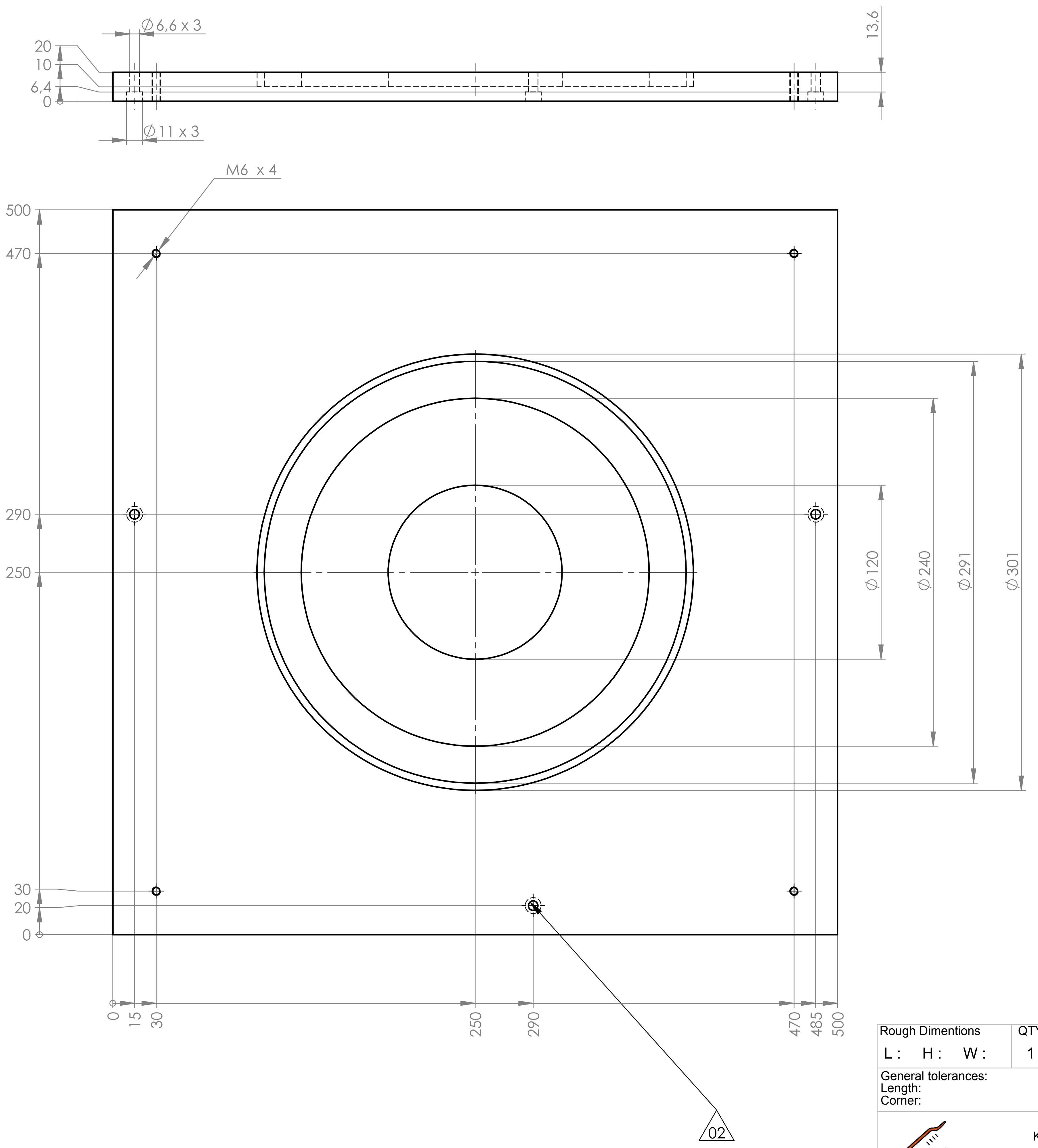

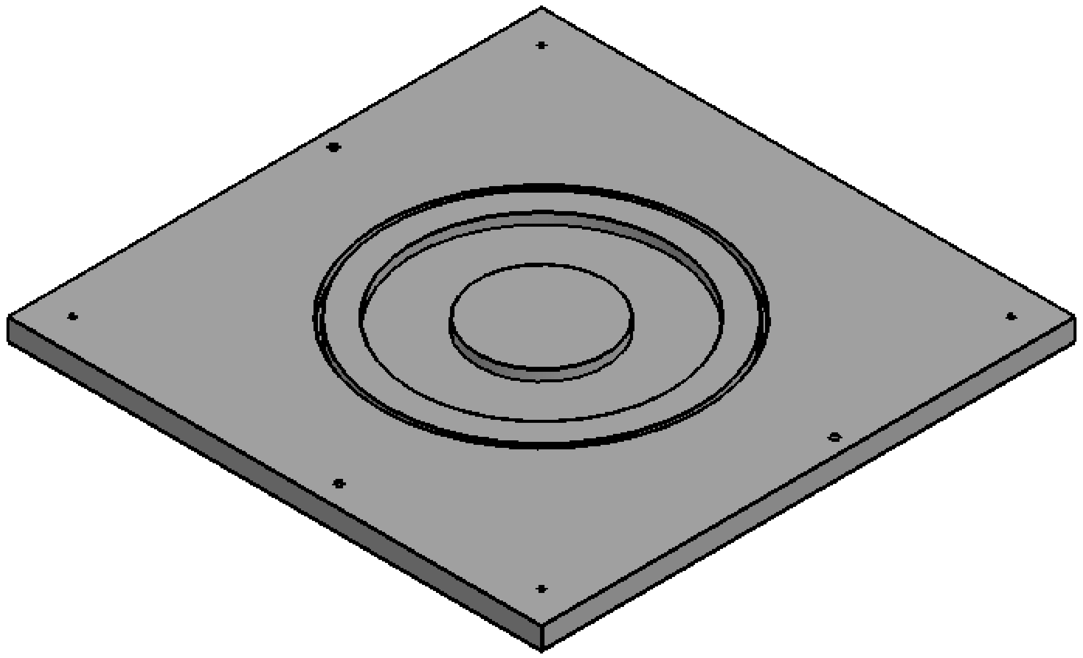

Rough Dimentions	QTY	Material			Drawn by		Scale
L : H : W :	1	POM (Wit)			Manu		1:3
General tolerances: Length: Corner:		Surface treatment	Hardness	Finishing	Created on 11-9-2014	Weight 6407.10 g	Sheetformat A3

PEIRA BVBA
Ketelaarstraat 8
2340 Beerse
www.peira.be
info@peira.be
Tel : +3214600800

Description
Bottem

Drawingnumber
PRA-09.006.01.101.00 02

Page
Blad 1

These drawings are and remain the property of Peira BVBA. Reuse or modification of the original drawings without written permission is prohibited. All rights reserved Peira BVBA.
